# A fractional differential equation model for the COVID-19 transmission by using the Caputo–Fabrizio derivative

**DOI:** 10.1186/s13662-020-02762-2

**Published:** 2020-06-18

**Authors:** Dumitru Baleanu, Hakimeh Mohammadi, Shahram Rezapour

**Affiliations:** 1 Department of Mathematics, Cankaya University, Ankara, Turkey; 2 Institute of Space Sciences, Magurele, Bucharest, Romania; 3 Department of Mathematics, Miandoab Branch, Islamic Azad University, Miandoab, Iran; 4 Department of Mathematics, Azarbaijan Shahid Madani University, Tabriz, Iran; 5 Department of Medical Research, China Medical University Hospital, China Medical University, Taichung, Taiwan

**Keywords:** 34A25, 34D20, 37M01, Fixed point, Homotopy analysis method, Mathematical model, Numerical simulation, Caputo–Fabrizio derivative

## Abstract

We present a fractional-order model for the COVID-19 transmission with Caputo–Fabrizio derivative. Using the homotopy analysis transform method (HATM), which combines the method of homotopy analysis and Laplace transform, we solve the problem and give approximate solution in convergent series. We prove the existence of a unique solution and the stability of the iteration approach by using fixed point theory. We also present numerical results to simulate virus transmission and compare the results with those of the Caputo derivative.

## Introduction

Corona viruses are a large family of viruses that have a distinctive corona or ‘crown’ of sugary-proteins, and because of their appearance, they were called corona viruses in 1960. Viruses that cause common cold diseases and fatal diseases, such as Middle East respiratory syndrome (MERS-CoV) and severe acute respiratory syndrome (SARS-CoV), are from the corona viruses family. Detailed investigations found that corona viruses are transmitted between animals and people, for instance, SARS-CoV and MERS-CoV were transmitted from civet cats and dromedary camels to humans, respectively. Also, several known corona viruses that have not yet infected humans are circulating in animals.

COVID-19, which was first identified in the Wuhan city, is a new strain that has not been previously identified in humans. Snakes or bats have been suspected as a potential source for the outbreak, though other experts currently consider this unlikely. Fever, cough, shortness of breath, and breathing difficulties are the initial symptoms of this infection. In the next steps, the infection can cause pneumonia, severe acute respiratory syndrome, kidney failure, and even death.

The study of disease dynamics is a dominating theme for many biologists and mathematicians (see, for example, [1–10]). It has been studied by many researchers that fractional extensions of mathematical models of integer order represent the natural fact in a very systematic way such as in the approach of Akbari et al. [11], Baleanu et al. [12–24], and Talaee et al. [25]. In this paper, we use the new fractional Caputo–Fabrizio derivative [26] to express the mathematical modeling for simulating the transmission of COVID-19. Recently, many works related to the fractional Caputo–Fabrizio derivative have been published (see, for example, [21, 23, 24, 27–30]). The Caputo–Fabrizio fractional derivative is also used to study the dynamics of diseases (see, for example, [31–34]). Mathematical models are used to simulate the transmission of corona virus (see, for example, [35, 36]). A mathematical model for the transmission of COVID-19 was presented by Chen et al. [37]. In this work, we investigate this model by using the Caputo–Fabrizio fractional derivative.

Now, we recall some fundamental notions. The Caputo fractional derivative of order *η* for a function *f* via integrable differentiations is defined by ${}^{C}D^{\eta }f(t)=\frac{1}{\varGamma (n-\eta )}\int _{0}^{t} \frac{f^{(n)}(s)}{(t-s)^{\eta -n+1}} \,ds$, where $n=[\eta ]+1$. Our second notion is a fractional derivative without singular kernel which was introduced by Caputo and Fabrizio in 2015 [26]. Let $b>a$, $f\in H^{1}(a,b)$, and $\eta \in (0,1)$. The Caputo–Fabrizio derivative of order *η* for a function *f* is defined by

$$ {}^{\mathrm{CF}}D^{\eta } f(t)=\frac{M(\eta )}{(1-\eta )} \int _{a}^{t} \exp \biggl(\frac{-\eta }{1-\eta }(t-s) \biggr)f'(s)\,ds, $$

where $t\geq 0$, $M(\eta )$ is a normalization function that depends on *η* and $M(0)=M(1)=1$. If $f \notin H^{1}(a,b)$ and $0<\eta <1$, this derivative can be presented for $f\in L^{1}(-\infty ,b)$ as

$$ {}^{\mathrm{CF}}D^{\eta } f(t)=\frac{\eta M(\eta )}{(1-\eta )} \int _{-\infty }^{b} \bigl(f(t)-f(s)\bigr)\exp \biggl( \frac{-\eta }{1-\eta }(t-s)\biggr)\,ds $$

(see [38]). Let $n\geq 1$ and $\eta \in (0,1)$. The fractional derivatives ${}^{\mathrm{CF}}D^{\eta +n}$ of order ${\eta +n}$ are defined by ${}^{\mathrm{CF}}D^{\eta +n} f(t):={ {}^{\mathrm{CF}}D^{\eta }(D^{n} f(t))}$ [28]. The Laplace transform of the Caputo–Fabrizio derivative is defined by $L[{}^{\mathrm{CF}}D^{(\eta +n)}f(t)](s)= \frac{s^{n+1}L[f(t)]-s^{n}f(0)-s^{n-1}f'(0)-\cdots-f^{(n)}(0)}{s+\eta (1-s)}$, where $0<\eta \leq 1$ and $M(\eta )=1$ [38].

The Riemann–Liouville fractional integral of order *η* with $\operatorname{Re} (\eta ) > 0$ is defined by $I^{\eta } f(t)= \frac{1}{\varGamma (\eta )} \int _{0}^{t} (t-s)^{\eta -1}f(s) \,ds$ [28]. The fractional integral of Caputo–Fabrizio is defined by ${}^{\mathrm{CF}}I^{\eta } f(t)=\frac{2(1-\eta )}{(2-\eta )M(\eta )} f(t)+ \frac{2\eta }{(2-\eta )M(\eta )}\int _{0}^{t} f(s)\,ds$ ($0<\eta <1$) [38]. The Sumudu transform is derived from the classical Fourier integral ([39–41]). Consider the set

$$ A=\biggl\{ F:\exists \lambda , k_{1} , k_{2} \geq 0, \bigl\vert F(t) \bigr\vert < \lambda \exp \biggl( \frac{t}{k_{j}}\biggr), t\in (-1)^{j} \times [0,\infty )\biggr\} . $$

The Sumudu transform of a function $f\in A$ is defined by

$$ F(u)=ST\bigl[f(t);u\bigr]= \frac{1}{u} \int _{0}^{\infty } \exp (-t/u)f(t)dt \quad \bigl[u\in (-k_{1},k_{2})\bigr] $$

for all $t\geq 0$, and the inverse Sumudu transform of $F(u)$ is denoted by $f(t)=ST^{-1}[F(u)]$ [40]. The Sumudu transform of the Caputo derivative is given by

$$ ST\bigl[{}^{c}D^{\eta }_{t} f(t);u \bigr]=u^{-\eta } \Biggl[F(u)- \sum_{i=0}^{m} u^{ \eta -i} \bigl[{}^{c} D^{\eta -i} f(t) \bigr]_{t=0}\Biggr], $$

where ($m-1< \eta \leq m$) [39]. Let *F* be a function such that its Caputo–Fabrizio fractional derivation exists. The Sumudu transform of *F* with Caputo–Fabrizio fractional derivative is defined by $ST({}^{\mathrm{CF}}_{0}D^{\eta }_{t})(F(t))=\frac{M(\eta )}{1-\eta +\eta u}[ST(F(t))-F(0)]$ [42].

## A mathematical model for the transmission of COVID-19 with Caputo–Fabrizio fractional derivative

Chen and colleagues have proposed a transmission network model to simulate possible transmission from the source of infection (possibly bats) to human infection [37]. They assumed that the virus was transmitted among the bats’ population, and then transmitted to an unknown host (probably wild animals). Then hosts were hunted and sent to the seafood market, which was defined as the reservoir or the virus. People exposed to the market got the risks of the infection. In the presented model, people were divided into five groups: susceptible people (S), exposed people (E), symptomatic infected people (I), asymptomatic infected people (A), and removed people (R) including recovered and dead people. COVID-19 in the reservoir was denoted as (W). This model was presented as follows:

$$ \textstyle\begin{cases} \frac{dS}{dt}= \varLambda -mS-\beta _{p}S(I+\kappa A)-\beta _{w}SW , \\ \frac{dE}{dt}=\beta _{p}S(I+\kappa A)+\beta _{w}SW-(1-\delta )\omega E- \delta \omega ^{\prime }E-mE , \\ \frac{dI}{dt}=(1-\delta )\omega E-(\gamma +m)I , \\ \frac{dA}{dt}=\delta \omega ^{\prime }_{p}E-(\gamma ^{\prime }+m)A, \\ \frac{dR}{dt}= \gamma I+\gamma ^{\prime }A-mR, \\ \frac{dW}{dt}=\mu I+\mu ^{\prime }A-\varepsilon W, \end{cases} $$

where

- $\varLambda =n\times N$, *N* refer to the total number of people and n is the birth rate,
- *m*: the death rate of people,
- $\beta _{p}$: the transmission rate from *I* to *S*,
- *κ*: the multiple of the transmissible of *A* to that of *I*,
- $\beta _{w}$: the transmission rate from *W* to *S*,
- *δ*: the proportion of asymptomatic infection rate of people
- $\frac{1}{\omega }$: the incubation period of people,
- $\frac{1}{\omega ^{\prime }}$: the latent period of people,
- $\frac{1}{\gamma }$: the infectious period of symptomatic infection of people,
- $\frac{1}{\gamma ^{\prime }}$: the infectious period of asymptomatic infection of people,
- *μ*: the shedding coefficients from *I* to *W*,
- $\mu ^{\prime }$: the shedding coefficients from *A* to *W*,
- $\frac{1}{\varepsilon }$: the lifetime of the virus in *W*.

Also, the initial conditions are $S(0)=S_{0}$, $E(0)=E_{0}$, $I(0)=I_{0}$, $A(0)=A_{0}$, $W(0)=W_{0}$.

We moderate the system by substituting the time derivative by the Caputo–Fabrizio fractional derivative in the Caputo sense [26]. With this change, the right- and left-hand sides will not have the same dimension. To solve this problem, we use an auxiliary parameter *ρ*, having the dimension of sec., to change the fractional operator so that the sides have the same dimension [43]. According to the explanation presented, the COVID-19 transmission fractional model for $t\geq 0$ and $\eta \in (0,1)$ is given as follows:

1 $$ \textstyle\begin{cases} \frac{1}{\rho ^{1-\eta }}\,{} ^{\mathrm{CF}}{D}^{\eta }_{t}S(t)= \varLambda -mS(t)- \beta _{p}S(t)(I(t)+\kappa A(t))-\beta _{w}S(t)W(t) , \\ \frac{1}{\rho ^{1-\eta }}\,{} ^{\mathrm{CF}}{D}^{\eta }_{t}E(t)=\beta _{p}S(t)(I(t)+ \kappa A(t))+\beta _{w}S(t)W(t) \\ \hphantom{\frac{1}{\rho ^{1-\eta }}\,{} ^{\mathrm{CF}}{D}^{\eta }_{t}E(t)={}}{} -(1-\delta )\omega E(t)-\delta \omega ^{\prime }E(t)-mE(t) , \\ \frac{1}{\rho ^{1-\eta }}\,{} ^{\mathrm{CF}}{D}^{\eta }_{t}I(t)=(1-\delta )\omega E(t)-( \gamma +m)I(t) , \\ \frac{1}{\rho ^{1-\eta }}\,{} ^{\mathrm{CF}}{D}^{\eta }_{t}A(t)=\delta \omega ^{\prime }_{p}E(t)-( \gamma ^{\prime }+m)A(t), \\ \frac{1}{\rho ^{1-\eta }}\,{} ^{\mathrm{CF}}{D}^{\eta }_{t}R(t)=\gamma I(t)+\gamma ^{\prime }A(t)-mR(t), \\ \frac{1}{\rho ^{1-\eta }}\,{} ^{\mathrm{CF}}{D}^{\eta }_{t}W(t)=\mu I(t)+\mu ^{\prime }A(t)- \varepsilon W(t), \end{cases} $$

where the initial conditions are $S(0)=S_{0}$, $E(0)=E_{0}$, $I(0)=I_{0}$, $A(0)=A_{0}$, $W(0)=W_{0}$. In the next section we investigate the existence and uniqueness of the solution for system (1) by fixed point theorem.

## Existence of a unique solution

In this section, we show that the system has a unique solution. For this purpose, employing the fractional integral operator due to Nieto and Losada [38] on the system (1), we obtain

$$ \textstyle\begin{cases} S(t)-S(0)=(\rho ^{1-\eta })\,{} ^{\mathrm{CF}}{I}^{\eta }_{t}[\varLambda -mS(t)-\beta _{p}S(t)(I(t)+ \kappa A(t))-\beta _{w}S(t)W(t)], \\ E(t)-E(0)=(\rho ^{1-\eta })\,{} ^{\mathrm{CF}}{I}^{\eta }_{t}[\beta _{p}S(t)(I(t)+ \kappa A(t))+\beta _{w}S(t)W(t) \\ \hphantom{E(t)-E(0)={}}{}-(1-\delta )\omega E(t)-\delta \omega ^{\prime }E(t)-mE(t)], \\ I(t)-I(0)=(\rho ^{1-\eta })\,{} ^{\mathrm{CF}}{I}^{\eta }_{t}[(1-\delta )\omega E(t)-( \gamma +m)I(t)], \\ A(t)-A(0)=(\rho ^{1-\eta })\,{} ^{\mathrm{CF}}{I}^{\eta }_{t}[\delta \omega ^{\prime }_{p}E(t)-( \gamma ^{\prime }+m)A(t)], \\ R(t)-R(0)=(\rho ^{1-\eta })\,{} ^{\mathrm{CF}}{I}^{\eta }_{t}[\gamma I(t)+\gamma ^{\prime }A(t)-mR(t)], \\ W(t)-W(0)=(\rho ^{1-\eta })\,{} ^{\mathrm{CF}}{I}^{\eta }_{t}[\mu I(t)+\mu ^{\prime }A(t)- \varepsilon W(t)]. \end{cases} $$

Using the definition of Caputo–Fabrizio fractional integral [38], we obtain

2 $$\begin{aligned}& S(t)-S(0)=\frac{2(1-\eta )\rho ^{1-\eta }}{(2-\eta )M(\eta )}\bigl\{ \varLambda -mS(t)-\beta _{p}S(t) \bigl(I(t)+\kappa A(t)\bigr)-\beta _{w}S(t)W(t)\bigr\} \\& \hphantom{S(t)-S(0)={}}{}+\frac{2\eta \rho ^{1-\eta }}{(2-\eta )M(\eta )} \int _{0}^{t} \bigl[\varLambda -mS(y)-\beta _{p}S(y) \bigl(I(y)+\kappa A(y)\bigr)-\beta _{w}S(y)W(y) \bigr]\,dy, \\& E(t)-E(0)=\frac{2(1-\eta )\rho ^{1-\eta }}{(2-\eta )M(\eta )}\bigl\{ \beta _{p}S(t) \bigl(I(t)+ \kappa A(t)\bigr)+\beta _{w}S(t)W(t) \\& \hphantom{E(t)-E(0)={}}{}-(1-\delta )\omega E(t)-\delta \omega ^{\prime }E(t)-mE(t) \bigr\} \\& \hphantom{E(t)-E(0)={}}{}+\frac{2\eta \rho ^{1-\eta }}{(2-\eta )M(\eta )} \int _{0}^{t} \bigl[\beta _{p}S(y) \bigl(I(y)+\kappa A(y)\bigr)+\beta _{w}S(y)W(y) \\& \hphantom{E(t)-E(0)=}{}-(1-\delta ) \omega E(y)-\delta \omega ^{\prime }E(y)-mE(y)\bigr]\,dy, \\& I(t)-I(0)=\frac{2(1-\eta )\rho ^{1-\eta }}{(2-\eta )M(\eta )}\bigl\{ (1- \delta )\omega E(t) -(\gamma +m)I(t) \bigr\} \\& \hphantom{I(t)-I(0)={}}{}+ \frac{2\eta \rho ^{1-\eta }}{(2-\eta )M(\eta )} \int _{0}^{t} \bigl[(1-\delta )\omega E(y)-( \gamma +m)I(y)\bigr]\,dy, \\& A(t)-A(0)=\frac{2(1-\eta )\rho ^{1-\eta }}{(2-\eta )M(\eta )}\bigl\{ \delta \omega ^{\prime }_{p}E(t)- \bigl(\gamma ^{\prime }+m\bigr)A(t)\bigr\} \\& \hphantom{A(t)-A(0)={}}{}+ \frac{2\eta \rho ^{1-\eta }}{(2-\eta )M(\eta )} \int _{0}^{t} \bigl[\delta \omega ^{\prime }_{p}E(t)-\bigl(\gamma ^{\prime }+m\bigr)A(t) \bigr]\,dy, \\& R(t)-R(0)=\frac{2(1-\eta )\rho ^{1-\eta }}{(2-\eta )M(\eta )}\bigl\{ \gamma I(t) +\gamma ^{\prime }A(t)-mR(t) \bigr\} \\& \hphantom{R(t)-R(0)={}}{}+ \frac{2\eta \rho ^{1-\eta }}{(2-\eta )M(\eta )} \int _{0}^{t} \bigl[\gamma I(y)+\gamma ^{\prime }A(y)-mR(y)\bigr]\,dy, \\& W(t)-W(0)=\frac{2(1-\eta )\rho ^{1-\eta }}{(2-\eta )M(\eta )}\bigl\{ \mu I(t)+ \mu ^{\prime }A(t)-\varepsilon W(t)\bigr\} \\& \hphantom{W(t)-W(0)={}}{}+ \frac{2\eta \rho ^{1-\eta }}{(2-\eta )M(\eta )} \int _{0}^{t} \bigl[\mu I(y)+\mu ^{\prime }A(y)-\varepsilon W(y)\bigr]\,dy. \end{aligned}$$

For convenience, we consider

$$ \textstyle\begin{cases} P_{1}(t,S)=\varLambda -mS(t)-\beta _{p}S(t)(I(t)+\kappa A(t))-\beta _{w}S(t)W(t), \\ P_{2}(t,E)=\beta _{p}S(t)(I(t)+\kappa A(t))+\beta _{w}S(t)W(t)-(1- \delta )\omega E(t)-\delta \omega ^{\prime }E(t)-mE(t), \\ P_{3}(t,I)=(1-\delta )\omega E(t)-(\gamma +m)I(t), \\ P_{4}(t,A)=\delta \omega ^{\prime }_{p}E(t)-(\gamma ^{\prime }+m)A(t), \\ P_{5}(t,R)=\gamma I(t)+\gamma ^{\prime }A(t)-mR(t), \\ P_{6}(t,W)=\mu I(t)+\mu ^{\prime }A(t)-\varepsilon W(t). \end{cases} $$

### Theorem 3.1

*The kernel*$P_{1}$*satisfies the Lipschitz condition and contraction if the following inequality holds*:

$$ 0< m+\beta _{p}l_{1}+\beta _{w}l_{2} \leq 1. $$

### Proof

Consider functions $S(t)$ and $S_{1}(t)$, then

$$\begin{aligned}& \bigl\Vert P_{1}\bigl(t,S(t)\bigr)-P_{1} \bigl(t,S_{1}(t)\bigr) \bigr\Vert \\& \quad = \bigl\Vert -m \bigl(S(t)-S_{1}(t)\bigr)-\beta _{p}I(t) \bigl(S(t)-S_{1}(t)\bigr)- \beta _{w}W(t) \bigl(S(t)-S_{1}(t)\bigr) \bigr\Vert \\& \quad \leq m \bigl\Vert S(t)-S_{1}(t) \bigr\Vert +\beta _{p} \bigl\Vert I(t) \bigr\Vert \bigl\Vert S(t)-S_{1}(t) \bigr\Vert +\beta _{w} \bigl\Vert W(t) \bigr\Vert \bigl\Vert S(t)-S_{1}(t) \bigr\Vert \\& \quad \leq \bigl(m+\beta _{p} \bigl\Vert I(t) \bigr\Vert +\beta _{w} \bigl\Vert W(t) \bigr\Vert \bigr) \bigl\Vert S(t)-S_{1}(t) \bigr\Vert \\& \quad \leq (m+\beta _{p}l_{1}+\beta _{w}l_{2}) \bigl\Vert S(t)-S_{1}(t) \bigr\Vert . \end{aligned}$$

Let $\lambda _{1}=m+\beta _{p}l_{1}+\beta _{w}l_{2}$, where $l_{1}=\|I(t)\|$ and $l_{2}=\|W(t)\|$ are bounded functions, then we have

$$ \bigl\Vert P_{1}\bigl(t,S(t)\bigr)-P_{1} \bigl(t,S_{1}(t)\bigr) \bigr\Vert \leq \lambda _{1} \bigl\Vert S(t)-S_{1}(t) \bigr\Vert . $$

Thus, the Lipschitz condition is fulfilled for $P_{1}$. In addition, if $0< m+\beta _{p}l_{1}+\beta _{w}l_{2}\leq 1$, then $P_{1}$ is a contraction. □

Similarly, $P_{2}$, $P_{3}$, $P_{4}$, $P_{5}$, $P_{6}$ satisfy the Lipschitz condition as follows:

$$\begin{aligned}& \bigl\Vert P_{2}\bigl(t,E(t)\bigr)-P_{2} \bigl(t,E_{1}(t)\bigr) \bigr\Vert \leq \lambda _{2} \bigl\Vert E(t)-E_{1}(t) \bigr\Vert , \\& \bigl\Vert P_{3}\bigl(t,I(t)\bigr)-P_{3} \bigl(t,I_{1}(t)\bigr) \bigr\Vert \leq \lambda _{3} \bigl\Vert I(t)-I_{1}(t) \bigr\Vert , \\& \bigl\Vert P_{4}\bigl(t,A(t)\bigr)-P_{4} \bigl(t,A_{1}(t)\bigr) \bigr\Vert \leq \lambda _{4} \bigl\Vert A(t)-A_{1}(t) \bigr\Vert , \\& \bigl\Vert P_{5}\bigl(t,R(t)\bigr)-P_{5} \bigl(t,R_{1}(t)\bigr) \bigr\Vert \leq \lambda _{5} \bigl\Vert R(t)-R_{1}(t) \bigr\Vert , \\& \bigl\Vert P_{6}\bigl(t,W(t)\bigr)-P_{6} \bigl(t,W_{1}(t)\bigr) \bigr\Vert \leq \lambda _{6} \bigl\Vert W(t)-W_{1}(t) \bigr\Vert . \end{aligned}$$

On consideration of $P_{1}$, $P_{2}$, $P_{3}$, $P_{4}$, $P_{5}$, $P_{6}$, we can write equation (2) as follows:

$$\begin{aligned}& S(t)=S(0)+\frac{2(1-\eta )\rho ^{1-\eta }}{(2-\eta )M(\eta )}P_{1}(t,S)+ \frac{2\eta \rho ^{1-\eta }}{(2-\eta )M(\eta )} \int _{0}^{t} \bigl(P_{1}(y,S)\bigr) \,dy, \\& E(t)=E(0)+\frac{2(1-\eta )\rho ^{1-\eta }}{(2-\eta )M(\eta )}P_{2}(t,E)+ \frac{2\eta \rho ^{1-\eta }}{(2-\eta )M(\eta )} \int _{0}^{t} \bigl(P_{2}(y,E) \bigr)\,dy, \\& I(t)=I(0)+\frac{2(1-\eta )\rho ^{1-\eta }}{(2-\eta )M(\eta )}P_{3}(t,I)+ \frac{2\eta \rho ^{1-\eta }}{(2-\eta )M(\eta )} \int _{0}^{t} \bigl(P_{3}(y,I) \bigr)\,dy, \\& A(t)=A(0)+\frac{2(1-\eta )\rho ^{1-\eta }}{(2-\eta )M(\eta )}P_{4}(t,A)+ \frac{2\eta \rho ^{1-\eta }}{(2-\eta )M(\eta )} \int _{0}^{t} \bigl(P_{4}(y,A) \bigr)\,dy, \\& R(t)=R(0)+\frac{2(1-\eta )\rho ^{1-\eta }}{(2-\eta )M(\eta )}P_{5}(t,R)+ \frac{2\eta \rho ^{1-\eta }}{(2-\eta )M(\eta )} \int _{0}^{t} \bigl(P_{5}(y,R) \bigr)\,dy, \\& W(t)=W(0)+\frac{2(1-\eta )\rho ^{1-\eta }}{(2-\eta )M(\eta )}P_{6}(t,W)+ \frac{2\eta \rho ^{1-\eta }}{(2-\eta )M(\eta )} \int _{0}^{t} \bigl(P_{6}(y,W) \bigr)\,dy. \end{aligned}$$

Thus, consider the following recursive formula:

$$\begin{aligned}& S_{n}(t)=\frac{2(1-\eta )\rho ^{1-\eta }}{(2-\eta )M(\eta )}P_{1}(t,S_{n-1})+ \frac{2\eta \rho ^{1-\eta }}{(2-\eta )M(\eta )} \int _{0}^{t} \bigl(P_{1}(y,S_{n-1}) \bigr)\,dy, \\& E_{n}(t)=\frac{2(1-\eta )\rho ^{1-\eta }}{(2-\eta )M(\eta )}P_{2}(t,E_{n-1})+ \frac{2\eta \rho ^{1-\eta }}{(2-\eta )M(\eta )} \int _{0}^{t} \bigl(P_{2}(y,E_{n-1}) \bigr)\,dy, \\& I_{n}(t)=\frac{2(1-\eta )\rho ^{1-\eta }}{(2-\eta )M(\eta )}P_{3}(t,I_{n-1})+ \frac{2\eta \rho ^{1-\eta }}{(2-\eta )M(\eta )} \int _{0}^{t} \bigl(P_{3}(y,I_{n-1}) \bigr)\,dy, \\& A_{n}(t)=\frac{2(1-\eta )\rho ^{1-\eta }}{(2-\eta )M(\eta )}P_{4}(t,A_{n-1})+ \frac{2\eta \rho ^{1-\eta }}{(2-\eta )M(\eta )} \int _{0}^{t} \bigl(P_{4}(y,A_{n-1}) \bigr)\,dy, \\& R_{n}(t)=\frac{2(1-\eta )\rho ^{1-\eta }}{(2-\eta )M(\eta )}P_{5}(t,R_{n-1})+ \frac{2\eta \rho ^{1-\eta }}{(2-\eta )M(\eta )} \int _{0}^{t} \bigl(P_{5}(y,R_{n-1}) \bigr)\,dy, \\& W_{n}(t)=\frac{2(1-\eta )\rho ^{1-\eta }}{(2-\eta )M(\eta )}P_{6}(t,W_{n-1})+ \frac{2\eta \rho ^{1-\eta }}{(2-\eta )M(\eta )} \int _{0}^{t} \bigl(P_{6}(y,W_{n-1}) \bigr)\,dy, \end{aligned}$$

where $S_{0}(t)=S(0)$, $E_{0}(t)=E(0)$, $I_{0}(t)=I(0)$, $A_{0}(t)=A(0)$, $R_{0}(t)=R(0)$, $W_{0}(t)=W(0)$.

Now, we consider

$$\begin{aligned}& H_{1n}=S_{n}(t)-S_{n-1}(t) \\& \hphantom{H_{1n}}= \frac{2(1-\eta )\rho ^{1-\eta }}{(2-\eta )M(\eta )}\bigl[P_{1}(t,S_{n-1})-P_{1}(t,S_{n-2}) \bigr] \\& \hphantom{H_{1n}={}}{}+\frac{2\eta \rho ^{1-\eta }}{(2-\eta )M(\eta )} \int _{0}^{t} \bigl[P_{1}(y,S_{n-1})-P_{1}(y,S_{n-2}) \bigr]\,dy, \\& H_{2n}=E_{n}(t)-E_{n-1}(t) \\& \hphantom{H_{2n}}= \frac{2(1-\eta )\rho ^{1-\eta }}{(2-\eta )M(\eta )}\bigl[P_{2}(t,E_{n-1})-P_{2}(t,E_{n-2}) \bigr] \\& \hphantom{H_{2n}={}}{}+\frac{2\eta \rho ^{1-\eta }}{(2-\eta )M(\eta )} \int _{0}^{t} \bigl[P_{2}(y,E_{n-1})-P_{2}(y,E_{n-2}) \bigr]\,dy, \\& H_{3n}=I_{n}(t)-I_{n-1}(t) \\& \hphantom{H_{3n}}= \frac{2(1-\eta )\rho ^{1-\eta }}{(2-\eta )M(\eta )}\bigl[P_{3}(t,I_{n-1})-P_{3}(t,I_{n-2}) \bigr] \\& \hphantom{H_{3n}={}}{}+\frac{2\eta \rho ^{1-\eta }}{(2-\eta )M(\eta )} \int _{0}^{t} \bigl[P_{3}(y,I_{n-1})-P_{3}(y,I_{n-2}) \bigr]\,dy, \\& H_{4n}=A_{n}(t)-A_{n-1}(t) \\& \hphantom{H_{4n}}= \frac{2(1-\eta )\rho ^{1-\eta }}{(2-\eta )M(\eta )}\bigl[P_{4}(t,A_{n-1})-P_{4}(t,A_{n-2}) \bigr] \\& \hphantom{H_{4n}={}}{}+\frac{2\eta \rho ^{1-\eta }}{(2-\eta )M(\eta )} \int _{0}^{t} \bigl[P_{4}(y,A_{n-1})-P_{4}(y,A_{n-2}) \bigr]\,dy, \\& H_{5n}=R_{n}(t)-R_{n-1}(t) \\& \hphantom{H_{5n}}= \frac{2(1-\eta )\rho ^{1-\eta }}{(2-\eta )M(\eta )}\bigl[P_{5}(t,R_{n-1})-P_{5}(t,R_{n-2}) \bigr] \\& \hphantom{H_{5n}={}}{}+\frac{2\eta \rho ^{1-\eta }}{(2-\eta )M(\eta )} \int _{0}^{t} \bigl[P_{5}(y,R_{n-1})-P_{5}(y,R_{n-2}) \bigr]\,dy, \\& H_{6n}=W_{n}(t)-W_{n-1}(t) \\& \hphantom{H_{6n}}= \frac{2(1-\eta )\rho ^{1-\eta }}{(2-\eta )M(\eta )}\bigl[P_{6}(t,W_{n-1})-P_{6}(t,W_{n-2}) \bigr] \\& \hphantom{H_{6n}={}}{}+\frac{2\eta \rho ^{1-\eta }}{(2-\eta )M(\eta )} \int _{0}^{t} \bigl[P_{6}(y,W_{n-1})-P_{6}(y,W_{n-2}) \bigr]\,dy. \end{aligned}$$

Given the above equations, one can write

3 $$ \begin{aligned} &S_{n}(t)=\sum_{j=0}^{n}H_{1j}(t),\qquad E_{n}(t)=\sum_{j=0}^{n}H_{2j}(t),\qquad I_{n}(t)= \sum_{j=0}^{n}H_{3j}(t), \\ &A_{n}(t)=\sum_{j=0}^{n}H_{4j}(t),\qquad R_{n}(t)=\sum_{j=0}^{n}H_{5j}(t),\qquad W_{n}(t)= \sum_{j=0}^{n}H_{6j}(t). \end{aligned} $$

According to $H_{1n}$’s definition and using the triangular inequality, we have

$$\begin{aligned} \bigl\Vert H_{1n}(t) \bigr\Vert =& \bigl\Vert S_{n}(t)-S_{n-1}(t) \bigr\Vert \\ =& \biggl\Vert \frac{2(1-\eta )\rho ^{1-\eta }}{(2-\eta )M(\eta )}\bigl[P_{1}(t,S_{n-1})-P_{1}(t,S_{n-2}) \bigr] \\ &{}+ \frac{2\eta \rho ^{1-\eta }}{(2-\eta )M(\eta )} \int _{0}^{t} \bigl[P_{1}(y,S_{n-1})-P_{1}(y,S_{n-2}) \bigr]\,dy \biggr\Vert \\ \leq& \frac{2(1-\eta )\rho ^{1-\eta }}{(2-\eta )M(\eta )} \bigl\Vert P_{1}(t,S_{n-1})-P_{1}(t,S_{n-2}) \bigr\Vert \\ &{}+\frac{2\eta \rho ^{1-\eta }}{(2-\eta )M(\eta )} \biggl\Vert \int _{0}^{t} \bigl[P_{1}(y,S_{n-1})-P_{1}(y,S_{n-2}) \bigr]\,dy \biggr\Vert . \end{aligned}$$

$P_{1}$ satisfies the Lipschitz condition, therefore

$$ \bigl\| S_{n}(t)-S_{n-1}(t)\bigr\| \leq \frac{2(1-\eta )\rho ^{1-\eta }}{(2-\eta )M(\eta )}\lambda _{1} \Vert S_{n-1}-S_{n-2} \Vert + \frac{2\eta \rho ^{1-\eta }}{(2-\eta )M(\eta )}\lambda _{1} \int _{0}^{t} \Vert S_{n-1}-S_{n-2} \Vert \,dy. $$

Thus we get

4 $$ \bigl\Vert H_{1n}(t) \bigr\Vert \leq \frac{2(1-\eta )\rho ^{1-\eta }}{(2-\eta )M(\eta )} \lambda _{1} \bigl\Vert H_{1n-1}(t) \bigr\Vert + \frac{2\eta \rho ^{1-\eta }}{(2-\eta )M(\eta )}\lambda _{1} \int _{0}^{t} \bigl\Vert H_{1n-1}(y) \bigr\Vert \,dy. $$

It can be shown that similar results are obtained for $H_{in},i=2,3,4,5,6$, as follows:

5 $$\begin{aligned}& \bigl\Vert H_{2n}(t) \bigr\Vert \leq \frac{2(1-\eta )\rho ^{1-\eta }}{(2-\eta )M(\eta )} \lambda _{2} \bigl\Vert H_{2n-1}(t) \bigr\Vert + \frac{2\eta \rho ^{1-\eta }}{(2-\eta )M(\eta )}\lambda _{2} \int _{0}^{t} \bigl\Vert H_{2n-1}(y) \bigr\Vert \,dy, \\& \bigl\Vert H_{3n}(t) \bigr\Vert \leq \frac{2(1-\eta )\rho ^{1-\eta }}{(2-\eta )M(\eta )} \lambda _{3} \bigl\Vert H_{3n-1}(t) \bigr\Vert + \frac{2\eta \rho ^{1-\eta }}{(2-\eta )M(\eta )}\lambda _{3} \int _{0}^{t} \bigl\Vert H_{3n-1}(y) \bigr\Vert \,dy, \\& \bigl\Vert H_{4n}(t) \bigr\Vert \leq \frac{2(1-\eta )\rho ^{1-\eta }}{(2-\eta )M(\eta )} \lambda _{4} \bigl\Vert H_{4n-1}(t) \bigr\Vert + \frac{2\eta \rho ^{1-\eta }}{(2-\eta )M(\eta )}\lambda _{4} \int _{0}^{t} \bigl\Vert H_{4n-1}(y) \bigr\Vert \,dy, \\& \bigl\Vert H_{5n}(t) \bigr\Vert \leq \frac{2(1-\eta )\rho ^{1-\eta }}{(2-\eta )M(\eta )} \lambda _{5} \bigl\Vert H_{5n-1}(t) \bigr\Vert + \frac{2\eta \rho ^{1-\eta }}{(2-\eta )M(\eta )}\lambda _{5} \int _{0}^{t} \bigl\Vert H_{5n-1}(y) \bigr\Vert \,dy, \\& \bigl\Vert H_{6n}(t) \bigr\Vert \leq \frac{2(1-\eta )\rho ^{1-\eta }}{(2-\eta )M(\eta )} \lambda _{6} \bigl\Vert H_{6n-1}(t) \bigr\Vert + \frac{2\eta \rho ^{1-\eta }}{(2-\eta )M(\eta )}\lambda _{6} \int _{0}^{t} \bigl\Vert H_{6n-1}(y) \bigr\Vert \,dy. \end{aligned}$$

According to the above result, we show that system (1) has a solution.

### Theorem 3.2

*The fractional COVID*-19 *model* (1) *has a system of solutions if there exist*$t_{i}$, $i=1,2,3,4,5,6$, *such that*

$$ \frac{2(1-\eta )\rho ^{1-\eta }}{(2-\eta )M(\eta )}\lambda _{i}+ \frac{2\eta \rho ^{1-\eta }}{(2-\eta )M(\eta )}\lambda _{i}t_{i}\leq 1. $$

### Proof

Assume that functions $S(t)$, $E(t)$, $I(t)$, $A(t)$, $R(t)$, $W(t)$ are bounded. We have shown that kernels $H_{in},i=1,2,3,4,5,6$, satisfy the Lipschitz condition. By using the recursive method and the results of (4) and (5), we obtain

$$\begin{aligned}& \bigl\Vert H_{1n}(t) \bigr\Vert \leq \bigl\Vert S(0) \bigr\Vert \biggl[ \frac{2(1-\eta )\rho ^{1-\eta }}{(2-\eta )M(\eta )}\lambda _{1}+ \frac{2\eta \rho ^{1-\eta }}{(2-\eta )M(\eta )}\lambda _{1}t\biggr]^{n}, \\& \bigl\Vert H_{2n}(t) \bigr\Vert \leq \bigl\Vert E(0) \bigr\Vert \biggl[ \frac{2(1-\eta )\rho ^{1-\eta }}{(2-\eta )M(\eta )}\lambda _{2}+ \frac{2\eta \rho ^{1-\eta }}{(2-\eta )M(\eta )}\lambda _{2}t\biggr]^{n}, \\& \bigl\Vert H_{3n}(t) \bigr\Vert \leq \bigl\Vert I(0) \bigr\Vert \biggl[ \frac{2(1-\eta )\rho ^{1-\eta }}{(2-\eta )M(\eta )}\lambda _{3}+ \frac{2\eta \rho ^{1-\eta }}{(2-\eta )M(\eta )}\lambda _{3}t\biggr]^{n}, \\& \bigl\Vert H_{4n}(t) \bigr\Vert \leq \bigl\Vert A(0) \bigr\Vert \biggl[ \frac{2(1-\eta )\rho ^{1-\eta }}{(2-\eta )M(\eta )}\lambda _{4}+ \frac{2\eta \rho ^{1-\eta }}{(2-\eta )M(\eta )}\lambda _{4}t\biggr]^{n}, \\& \bigl\Vert H_{5n}(t) \bigr\Vert \leq \bigl\Vert R(0) \bigr\Vert \biggl[ \frac{2(1-\eta )\rho ^{1-\eta }}{(2-\eta )M(\eta )}\lambda _{5}+ \frac{2\eta \rho ^{1-\eta }}{(2-\eta )M(\eta )}\lambda _{5}t\biggr]^{n}, \\& \bigl\Vert H_{6n}(t) \bigr\Vert \leq \bigl\Vert W(0) \bigr\Vert \biggl[ \frac{2(1-\eta )\rho ^{1-\eta }}{(2-\eta )M(\eta )}\lambda _{6}+ \frac{2\eta \rho ^{1-\eta }}{(2-\eta )M(\eta )}\lambda _{6}t\biggr]^{n}. \end{aligned}$$

Thus, functions (3) exist and are smooth. We claim that the above functions are the solutions of system (1). To prove this claim, we assume

$$\begin{aligned}& S(t)-S(0)=H_{1n}(t)-G_{1n}(t),\qquad E(t)-E(0)=H_{2n}(t)-G_{2n}(t), \\& I(t)-I(0)=H_{3n}(t)-G_{3n}(t),\qquad A(t)-A(0)=H_{4n}(t)-G_{4n}(t), \\& R(t)-R(0)=H_{5n}(t)-G_{5n}(t),\qquad W(t)-W(0)=H_{6n}(t)-G_{6n}(t). \end{aligned}$$

We have

$$\begin{aligned} \bigl\Vert G_{1n}(t) \bigr\Vert =& \biggl\Vert \frac{2(1-\eta )\rho ^{1-\eta }}{(2-\eta )M(\eta )}\bigl[P_{1}(t,S)-P_{1}(t,S_{n-1}) \bigr] \\ &{}+ \frac{2\eta \rho ^{1-\eta }}{(2-\eta )M(\eta )} \int _{0}^{t} \bigl[P_{1}(y,S)-P(y,S_{n-1}) \bigr]\,dy \biggr\Vert \\ \leq& \frac{2(1-\eta )\rho ^{1-\eta }}{(2-\eta )M(\eta )} \bigl\Vert P_{1}(t,S)-P_{1}(t,S_{n-1}) \bigr\Vert \\ &{}+\frac{2\eta \rho ^{1-\eta }}{(2-\eta )M(\eta )} \int _{0}^{t} \bigl\Vert P_{1}(y,S)-P(y,S_{n-1}) \bigr\Vert \,dy \\ \leq& \frac{2(1-\eta )\rho ^{1-\eta }}{(2-\eta )M(\eta )}\lambda _{1} \Vert S-S_{n-1} \Vert +\frac{2\eta \rho ^{1-\eta }}{(2-\eta )M(\eta )}\lambda _{1} \Vert S-S_{n-1} \Vert t. \end{aligned}$$

By repeating this process, we obtain

$$ \bigl\Vert G_{1n}(t) \bigr\Vert \leq \biggl[ \frac{2(1-\eta )\rho ^{1-\eta }}{(2-\eta )M(\eta )}+ \frac{2\eta \rho ^{1-\eta }}{(2-\eta )M(\eta )}t\biggr]^{n+1} \lambda _{1}^{n+1}q. $$

By taking limit on recent equation as *n* tends to infinity, we obtain $\|G_{1n}(t)\|\rightarrow 0$. By the same way, we get $\|G_{in}(t)\|\rightarrow 0$, $i=2,3,4,5,6$, and this completes the proof. □

To prove the uniqueness of solution, we assume that system (1) has another solution such as $S_{1}$, $E_{1}$, $I_{1}$, $A_{1}$, $R_{1}$, $W_{1}$. Then

$$\begin{aligned}& \bigl\Vert S(t)-S_{1}(t) \bigr\Vert \\& \quad = \biggl\| \frac{2(1-\eta )\rho ^{1-\eta }}{(2-\eta )M(\eta )}\bigl(P_{1}(t,S)-P_{1}(t,S_{1})\bigr)+ \frac{2\eta \rho ^{1-\eta }}{(2-\eta )M(\eta )} \int _{0}^{t} \bigl(P_{1}(y,S)-P_{1}(y,S_{1})\bigr) \,dy\biggr\| \\& \quad \leq \frac{2(1-\eta )\rho ^{1-\eta }}{(2-\eta )M(\eta )}\bigl\| P_{1}(t,S)-P_{1}(t,S_{1}) \bigr\Vert +\frac{2\eta \rho ^{1-\eta }}{(2-\eta )M(\eta )} \int _{0}^{t} \bigl\| P_{1}(y,S)-P_{1}(y,S_{1}) \bigr\| \,dy. \end{aligned}$$

According to the Lipschitz condition of *S*, we get

$$ \bigl\Vert S(t)-S_{1}(t) \bigr\Vert \leq \frac{2(1-\eta )\rho ^{1-\eta }}{(2-\eta )M(\eta )} \lambda _{1} \bigl\Vert S(t)-S_{1}(t) \bigr\Vert + \frac{2\eta \rho ^{1-\eta }}{(2-\eta )M(\eta )}\lambda _{1}t \bigl\Vert S(t)-S_{1}(t) \bigr\Vert . $$

Thus

6 $$ \bigl\Vert S(t)-S_{1}(t) \bigr\Vert \biggl(1- \frac{2(1-\eta )\rho ^{1-\eta }}{(2-\eta )M(\eta )}\lambda _{1}- \frac{2\eta \rho ^{1-\eta }}{(2-\eta )M(\eta )}\lambda _{1}t\biggr)\leq 0. $$

### Theorem 3.3

*The solution of COVID*-19 *fractional model* (1) *is unique if the following condition holds*:

7 $$ \biggl(1-\frac{2(1-\eta )\rho ^{1-\eta }}{(2-\eta )M(\eta )}\lambda _{1}- \frac{2\eta \rho ^{1-\eta }}{(2-\eta )M(\eta )}\lambda _{1}t\biggr)\geq 0. $$

### Proof

From condition (7) and equation (6), we conclude that

$$ \bigl\Vert S(t)-S_{1}(t) \bigr\Vert \biggl(1- \frac{2(1-\eta )\rho ^{1-\eta }}{(2-\eta )M(\eta )}\lambda _{1}- \frac{2\eta \rho ^{1-\eta }}{(2-\eta )M(\eta )}\lambda _{1}t\biggr)=0. $$

So $\|S(t)-S_{1}(t)\|=0$, then $S(t)=S_{1}(t)$. In the same way, we can show that

$$ E(t)=E_{1}(t),\qquad I(t)=I_{1}(t),\qquad A(t)=A_{1}(t),\qquad R(t)=R_{1}(t),\qquad W(t)=W_{1}(t). $$

The proof is complete. □

## Stability analysis by fixed point theory

Using the Sumudu transform, we obtain a special solution to the COVID-19 model and then prove the stability of the iterative method using fixed point theory. At first, we apply the Sumudu transform on both sides of equations in model (1), then

$$ \textstyle\begin{cases} ST(\frac{1}{\rho ^{1-\eta }}\,{} ^{\mathrm{CF}}{D}^{\eta }_{t}S(t))=ST( \varLambda -mS(t)- \beta _{p}S(t)(I(t)+\kappa A(t))-\beta _{w}S(t)W(t)) , \\ ST(\frac{1}{\rho ^{1-\eta }}\,{} ^{\mathrm{CF}}{D}^{\eta }_{t}E(t))=ST(\beta _{p}S(t)(I(t)+ \kappa A(t))+\beta _{w}S(t)W(t) \\ \hphantom{ST(\frac{1}{\rho ^{1-\eta }}\,{} ^{\mathrm{CF}}{D}^{\eta }_{t}E(t))={}}{}-(1-\delta )\omega E(t)-\delta \omega ^{\prime }E(t)-mE(t)) , \\ ST(\frac{1}{\rho ^{1-\eta }}\,{} ^{\mathrm{CF}}{D}^{\eta }_{t}I(t))=ST((1-\delta ) \omega E(t)-(\gamma +m)I(t)) , \\ ST(\frac{1}{\rho ^{1-\eta }}\,{} ^{\mathrm{CF}}{D}^{\eta }_{t}A(t))=ST(\delta \omega ^{\prime }_{p}E(t)-(\gamma ^{\prime }+m)A(t)), \\ ST(\frac{1}{\rho ^{1-\eta }}\,{} ^{\mathrm{CF}}{D}^{\eta }_{t}R(t))=ST(\gamma I(t)+ \gamma ^{\prime }A(t)-mR(t)), \\ ST(\frac{1}{\rho ^{1-\eta }}\,{} ^{\mathrm{CF}}{D}^{\eta }_{t}W(t))=ST(\mu I(t)+ \mu ^{\prime }A(t)-\varepsilon W(t)). \end{cases} $$

We conclude from the Sumudu transform definition of the Caputo–Fabrizio derivative the following:

$$ \textstyle\begin{cases} \frac{M(\eta )}{1-\eta +\eta u}(ST(S(t))-S(0))=\rho ^{1-\eta } ST( \varLambda -mS(t)-\beta _{p}S(t)(I(t)+\kappa A(t))-\beta _{w}S(t)W(t)), \\ \frac{M(\eta )}{1-\eta +\eta u}(ST(E(t))-E(0))=\rho ^{1-\eta } ST( \beta _{p}S(t)(I(t)+\kappa A(t))+\beta _{w}S(t)W(t)-(1-\delta ) \omega E(t) \\ \hphantom{\frac{M(\eta )}{1-\eta +\eta u}(ST(E(t))-E(0))={}}{}-\delta \omega ^{\prime }E(t)-mE(t)), \\ \frac{M(\eta )}{1-\eta +\eta u}(ST(I(t))-I(0))=\rho ^{1-\eta } ST((1- \delta )\omega E(t)-(\gamma +m)I(t)), \\ \frac{M(\eta )}{1-\eta +\eta u}(ST(A(t))-A(0))= \rho ^{1-\eta } ST( \delta \omega ^{\prime }_{p}E(t)-(\gamma ^{\prime }+m)A(t)), \\ \frac{M(\eta )}{1-\eta +\eta u}(ST(R(t))-R(0))=\rho ^{1-\eta } ST( \gamma I(t)+\gamma ^{\prime }A(t)-mR(t)), \\ \frac{M(\eta )}{1-\eta +\eta u}(ST(W(t))-W(0))=\rho ^{1-\eta } ST( \mu I(t)+\mu ^{\prime }A(t)-\varepsilon W(t)). \end{cases} $$

If we rearrange the above inequalities, then

$$ \textstyle\begin{cases} ST(S(t))=S(0)+\frac{1-\eta +\eta u}{M(\eta )} \rho ^{1-\eta } ST[ \varLambda -mS(t)-\beta _{p}S(t)(I(t)+\kappa A(t))-\beta _{w}S(t)W(t)], \\ ST(E(t))=E(0)+\frac{1-\eta +\eta u}{M(\eta )} \rho ^{1-\eta } ST[ \beta _{p}S(t)(I(t)+\kappa A(t))+\beta _{w}S(t)W(t)-(1-\delta ) \omega E(t) \\ \hphantom{ST(E(t))={}}{}-\delta \omega ^{\prime }E(t)-mE(t)], \\ ST(I(t))=I(0)+\frac{1-\eta +\eta u}{M(\eta )} \rho ^{1-\eta } ST[(1- \delta )\omega E(t)-(\gamma +m)I(t)], \\ ST(A(t))=A(0)+\frac{1-\eta +\eta u}{M(\eta )} \rho ^{1-\eta } ST[ \delta \omega ^{\prime }_{p}E(t)-(\gamma ^{\prime }+m)A(t)], \\ ST(R(t))=R(0)+\frac{1-\eta +\eta u}{M(\eta )} \rho ^{1-\eta } ST[ \gamma I(t)+\gamma ^{\prime }A(t)-mR(t)], \\ ST(W(t))=W(0)+\frac{1-\eta +\eta u}{M(\eta )} \rho ^{1-\eta } ST[\mu I(t)+ \mu ^{\prime }A(t)-\varepsilon W(t)]. \end{cases} $$

We obtain

8 $$ \textstyle\begin{cases} S_{n+1}(t)=S_{n}(0)+ST^{-1}\{\frac{1-\eta +\eta u}{M(\eta )} \rho ^{1- \eta } ST[\varLambda -mS_{n}(t)-\beta _{p}S_{n}(t)(I_{n}(t)+\kappa A_{n}(t)) \\ \hphantom{S_{n+1}(t)={}}{}-\beta _{w}S_{n}(t)W_{n}(t)]\}, \\ E_{n+1}(t)=E_{n}(0)+ST^{-1}\{\frac{1-\eta +\eta u}{M(\eta )} \rho ^{1- \eta } ST[\beta _{p}S_{n}(t)(I_{n}(t)+\kappa A_{n}(t))+\beta _{w}S_{n}(t)W_{n}(t) \\ \hphantom{E_{n+1}(t)={}}{}-(1-\delta )\omega E_{n}(t)-\delta \omega ^{\prime }E_{n}(t)-mE_{n}(t)]\}, \\ I_{n+1}(t)=I_{n}(0)+ST^{-1}\{\frac{1-\eta +\eta u}{M(\eta )} \rho ^{1- \eta } ST[(1-\delta )\omega E_{n}(t)-(\gamma +m)I_{n}(t)]\}, \\ A_{n+1}(t)=A_{n}(0)+ST^{-1}\{\frac{1-\eta +\eta u}{M(\eta )} \rho ^{1- \eta } ST[\delta \omega ^{\prime }_{p}E_{n}(t)-(\gamma ^{\prime }+m)A_{n}(t)]\}, \\ R_{n+1}(t)=R_{n}(0)+ST^{-1}\{\frac{1-\eta +\eta u}{M(\eta )} \rho ^{1- \eta } ST[\gamma I_{n}(t)+\gamma ^{\prime }A_{n}(t)-mR_{n}(t)]\}, \\ W_{n+1}(t)=W_{n}(0)+ST^{-1}\{\frac{1-\eta +\eta u}{M(\eta )} \rho ^{1- \eta } ST[\mu I_{n}(t)+\mu ^{\prime }A_{n}(t)-\varepsilon W_{n}(t)]\}. \end{cases} $$

The approximate solution of system (1) is as follows:

$$\begin{aligned}& S(t)=\lim_{n\rightarrow \infty }S_{n}(t), \qquad E(t)=\lim _{n\rightarrow \infty }E_{n}(t), \qquad I(t)=\lim_{n\rightarrow \infty }I_{n}(t), \\& A(t)=\lim_{n\rightarrow \infty }A_{n}(t),\qquad R(t)=\lim _{n\rightarrow \infty }R_{n}(t),\qquad W(t)=\lim_{n\rightarrow \infty }W_{n}(t). \end{aligned}$$

### Stability analysis of iteration method

Consider the Banach space $(G,\|\cdot\|)$, a self-map *T* on *G*, and the recursive method $q_{n+1}=\phi (T, q_{n})$. Assume that $\varUpsilon (T)$ is the fixed point set of *T* which $\varUpsilon (T)\neq \emptyset $ and $\lim_{n\rightarrow \infty }q_{n}=q\in \varUpsilon (T)$. Suppose that $\{t_{n}\}\subset \varUpsilon $ and $r_{n}=\| t_{n+1}-\phi (T, t_{n})\|$. If $\lim_{n\rightarrow \infty }r_{n}=0$ implies that $\lim_{n\rightarrow \infty }t_{n}=q$, then the recursive procedure $q_{n+1}=\phi (T,q_{n})$ is *T*-stable. Suppose that our sequence $\{t_{n}\}$ has an upper boundary. If Picard’s iteration $q_{n+1}=Tq_{n}$ is satisfied in all these conditions, then $q_{n+1}=Tq_{n}$ is *T*-stable.

#### Theorem 4.1

([44])

*Let*$(G , \|\cdot\|)$*be a Banach space andTbe a self*-*map ofGsatisfying*

$$ \Vert T_{x}-T_{y} \Vert \leq B \Vert x-T_{x} \Vert + b \Vert x-y \Vert $$

*for all*$x,y\in G$*where*$B\geq 0$*and*$0\leq b <1$. *Suppose thatTis PicardT*-*stable*.

According to (8), the fractional model of COVID-19 (1) is connected with the subsequent iterative formula. Now consider the following theorem.

#### Theorem 4.2

*Suppose thatTis a self*-*map defined as follows*:

$$ \textstyle\begin{cases} T(S_{n}(t))=S_{n+1}(t) \\ \hphantom{T(S_{n}(t))}=S_{n}(t)+ST^{-1}\{ \frac{1-\eta +\eta u}{M(\eta )} \rho ^{1-\eta } ST[\varLambda -mS_{n}(t) \\ \hphantom{T(S_{n}(t))={}}{}-\beta _{p}S_{n}(t)(I_{n}(t)+\kappa A_{n}(t))-\beta _{w}S_{n}(t)W_{n}(t)] \}, \\ T(E_{n}(t))=E_{n+1}(t) \\ \hphantom{T(E_{n}(t))}=E_{n}(t)+ST^{-1}\{ \frac{1-\eta +\eta u}{M(\eta )} \rho ^{1-\eta } ST[\beta _{p}S_{n}(t)(I_{n}(t)+ \kappa A_{n}(t)) \\ \hphantom{T(E_{n}(t))={}}{}+\beta _{w}S_{n}(t)W_{n}(t)-(1-\delta )\omega E_{n}(t)-\delta \omega ^{\prime }E_{n}(t)-mE_{n}(t)] \}, \\ T(I_{n}(t))=I_{n+1}(t)=I_{n}(t)+ST^{-1}\{ \frac{1-\eta +\eta u}{M(\eta )} \rho ^{1-\eta } ST[(1-\delta )\omega E_{n}(t)-( \gamma +m)I_{n}(t)]\}, \\ T(A_{n}(t))=A_{n+1}(t)=A_{n}(t)+ST^{-1}\{ \frac{1-\eta +\eta u}{M(\eta )} \rho ^{1-\eta } ST[\delta \omega ^{\prime }_{p}E_{n}(t)-( \gamma ^{\prime }+m)A_{n}(t)]\}, \\ T(R_{n}(t))=R_{n+1}(t)=R_{n}(t)+ST^{-1}\{ \frac{1-\eta +\eta u}{M(\eta )} \rho ^{1-\eta } ST[\gamma I_{n}(t)+ \gamma ^{\prime }A_{n}(t)-mR_{n}(t)]\}, \\ T(W_{n}(t))=W_{n+1}(t)=W_{n}(t)+ST^{-1}\{ \frac{1-\eta +\eta u}{M(\eta )} \rho ^{1-\eta } ST[\mu I_{n}(t)+\mu ^{\prime }A_{n}(t)- \varepsilon W_{n}(t)]\}. \end{cases} $$

*This iterative recursive isT*-*stable in*$L^{1}(a,b)$*if the following conditions are achieved*:

$$ \textstyle\begin{cases} (1-(m+\beta _{p}M_{3}+\beta _{p}M_{4}+\beta _{w}M_{6})f_{1}(\eta )- \beta _{p}M_{1}f_{2}(\eta )-\beta _{p}\kappa M_{1}f_{4}(\eta )-\beta _{w}M_{1}f_{4}( \eta ))< 1, \\ (1+\beta _{p}M_{1}f_{5}(\eta )+(\beta _{p}M_{3}+\beta _{p}\kappa M_{4}+ \beta _{w}M_{6})f_{6}(\eta )+\beta _{p}\kappa M_{1}f_{7}(\eta )+ \beta _{w}M_{1}f_{8}(\eta ) \\ \quad {}-((1-\delta )m+\delta \omega ^{\prime } +m)f_{9}(\eta ))< 1, \\ (1+(1-\delta )\omega f_{10}(\eta )-(\gamma +m)f_{11}(\eta ))< 1, \\ (1+\delta \omega ^{\prime }_{p}f_{12}(\eta )-(\gamma ^{\prime }+m)f_{13}(\eta ))< 1, \\ (1+\gamma f_{14}(\eta )+\gamma ^{\prime } f_{15}(\eta )-mf_{16}(\eta ))< 1, \\ (1+\mu f_{17}(\eta )+\mu ^{\prime } f_{18}(\eta )-\varepsilon f_{19}(\eta ))< 1. \end{cases} $$

#### Proof

To prove that *T* has a fixed point, we compute the following inequalities for $(i,j)\in N\times N$:

$$\begin{aligned}& T\bigl(S_{i}(t)\bigr)-T\bigl(S_{j}(t) \bigr) \\& \quad =S_{i}(t)-S_{j}(t)+ST^{-1}\biggl\{ \frac{1-\eta +\eta u}{M(\eta )} \rho ^{1-\eta } ST\bigl[\bigl(\varLambda -mS_{i}(t)-\beta _{p}S_{i}(t) \bigl(I_{i}(t)+\kappa A_{i}(t)\bigr) \\& \qquad {}-\beta _{w}S_{i}(t)W_{i}(t) \bigr)-\bigl( \varLambda -mS_{j}(t)-\beta _{p}S_{j}(t) \bigl(I_{j}(t)+\kappa A_{j}(t)\bigr)- \beta _{w}S_{j}(t)W_{j}(t)\bigr)\bigr]\biggr\} \\& \quad =\bigl(S_{i}(t)-S_{j}(t)\bigr) \\& \qquad {}+ST^{-1} \biggl\{ \frac{1-\eta +\eta u}{M(\eta )} \rho ^{1- \eta } ST\bigl[-\bigl(m+\beta _{p}I_{j}(t)+\beta _{p}\kappa A_{j}(t)+\beta _{w}W_{j}(t)\bigr) \bigl(S_{i}(t)-S_{j}(t)\bigr) \\& \qquad {}-\beta _{p}S_{i}(t) \bigl(I_{i}(t)-I_{j}(t) \bigr)-\beta _{p}\kappa S_{i}(t) \bigl(A_{i}(t)-A_{j}(t) \bigr)- \beta _{w}S_{i}(t) \bigl(W_{i}(t)-W_{j}(t) \bigr)\bigr]\biggr\} . \end{aligned}$$

By applying norm on both sides, we obtain

9 $$\begin{aligned}& \bigl\Vert T\bigl(S_{i}(t)\bigr)-T\bigl(S_{j}(t) \bigr) \bigr\Vert \\& \quad = \biggl\Vert \bigl(S_{i}(t)-S_{j}(t) \bigr) \\& \qquad {}+ST^{-1}\biggl\{ \frac{1-\eta +\eta u}{M(\eta )} \rho ^{1- \eta } ST \bigl[-\bigl(m+\beta _{p}I_{j}(t)+\beta _{p}\kappa A_{j}(t)+\beta _{w}W_{j}(t) \bigr) \bigl(S_{i}(t)-S_{j}(t)\bigr) \\& \qquad {}-\beta _{p}S_{i}(t) \bigl(I_{i}(t)-I_{j}(t) \bigr)-\beta _{p}\kappa S_{i}(t) \bigl(A_{i}(t)-A_{j}(t) \bigr)- \beta _{w}S_{i}(t) \bigl(W_{i}(t)-W_{j}(t) \bigr)\bigr]\biggr\} \biggr\Vert \\& \quad \leq \bigl\Vert S_{i}(t)-S_{j}(t) \bigr\Vert \\& \qquad {}+ST^{-1}\biggl\{ \frac{1-\eta +\eta u}{M(\eta )} \rho ^{1-\eta } ST\bigl[ \bigl\Vert -\bigl(m+\beta _{p}I_{j}(t)+\beta _{p}\kappa A_{j}(t)+ \beta _{w}W_{j}(t) \bigr) \bigl(S_{i}(t)-S_{j}(t)\bigr) \bigr\Vert \\& \qquad {} + \bigl\Vert -\beta _{p}S_{i}(t) \bigl(I_{i}(t)-I_{j}(t)\bigr) \bigr\Vert + \bigl\Vert -\beta _{p}\kappa S_{i}(t) \bigl(A_{i}(t)-A_{j}(t) \bigr) \bigr\Vert \\& \qquad {}+ \bigl\Vert -\beta _{w}S_{i}(t) \bigl(W_{i}(t)-W_{j}(t)\bigr) \bigr\Vert \bigr]\biggr\} . \end{aligned}$$

Since the solutions have the same roles, we can consider

10 $$\begin{aligned} \bigl\Vert S_{i}(t)-S_{j}(t) \bigr\Vert &\cong \bigl\Vert E_{i}(t)-E_{j}(t) \bigr\Vert \cong \bigl\Vert I_{i}(t)-I_{j}(t) \bigr\Vert \cong \bigl\Vert A_{i}(t)-A_{j}(t) \bigr\Vert \\ & \cong \bigl\Vert R_{n}(t)-R_{m}(t) \bigr\Vert \cong \bigl\Vert R_{n}(t)-R_{m}(t) \bigr\Vert . \end{aligned}$$

From equations (9) and (10), we get

11 $$\begin{aligned}& \bigl\Vert T\bigl(S_{i}(t)\bigr)-T\bigl(S_{j}(t) \bigr) \bigr\Vert \\& \quad \leq \bigl\Vert S_{i}(t)-S_{j}(t) \bigr\Vert \\& \qquad {}+ST^{-1}\biggl\{ \frac{1-\eta +\eta u}{M(\eta )} \rho ^{1-\eta } ST\bigl[ \bigl\Vert -\bigl(m+\beta _{p}I_{j}(t)+\beta _{p}\kappa A_{j}(t)+ \beta _{w}W_{j}(t) \bigr) \bigl(S_{i}(t)-S_{j}(t)\bigr) \bigr\Vert \\& \qquad {}+ \bigl\Vert -\beta _{p}S_{i}(t) \bigl(S_{i}(t)-S_{j}(t)\bigr) \bigr\Vert + \bigl\Vert -\beta _{p}\kappa S_{i}(t) \bigl(S_{i}(t)-S_{j}(t) \bigr) \bigr\Vert \\& \qquad {}+ \bigl\Vert -\beta _{w}S_{i}(t) \bigl(S_{i}(t)-S_{j}(t)\bigr) \bigr\Vert \bigr]\biggr\} . \end{aligned}$$

$S_{i}$, $E_{i}$, $I_{i}$, $A_{i}$, $R_{i}$, $W_{i}$ are bounded because they are convergent sequences, then for all *t* there exist $M_{i}$, $i=1,2,3,4,5,6$, such that

12 $$ \begin{aligned} &\Vert S_{i} \Vert < M_{1},\qquad \Vert E_{i} \Vert < M_{2},\qquad \Vert I_{i} \Vert < M_{3},\qquad \Vert A_{i} \Vert < M_{4}, \\ & \Vert R_{i} \Vert < M_{5},\qquad \Vert W_{i} \Vert < M_{6}, \quad (i,j)\in N \times N. \end{aligned} $$

From equations (11) and (12), we get

13 $$\begin{aligned}& \bigl\| T\bigl(S_{i}(t)\bigr)-T\bigl(S_{j}(t)\bigr)\bigr\| \\& \quad \leq \bigl[1-(m+\beta _{p}M_{3}+\beta _{p}M_{4}+\beta _{w}M_{6})f_{1}( \eta )-\beta _{p}M_{1}f_{2}(\eta )-\beta _{p}\kappa M_{1}f_{4}(\eta )- \beta _{w}M_{1}f_{4}(\eta )\bigr] \\& \qquad {}\times \bigl\Vert S_{i}(t)-S_{j}(t) \bigr\Vert , \end{aligned}$$

where $f_{i}$ are functions from $ST^{-1}[\frac{1-\eta +\eta u}{M(\eta )} \rho ^{1-\eta }ST[*]]$. Similarly, we will obtain

14 $$ \textstyle\begin{cases} \|T(E_{i}(t)-T(E_{j}(t))\| \\ \quad \leq [1+\beta _{p}M_{1}f_{5}(\eta )+( \beta _{p}M_{3}+\beta _{p}\kappa M_{4}+\beta _{w}M_{6})f_{6}(\eta )+ \beta _{p}\kappa M_{1}f_{7}(\eta ) \\ \qquad {}+\beta _{w}M_{1}f_{8}(\eta )-((1-\delta )m+\delta \omega ^{\prime } +m)f_{9}( \eta )] \Vert E_{i}(t)-E_{j}(t) \Vert , \\ \|T(I_{i}(t)-T(I_{j}(t))\| \leq [1+(1-\delta )\omega f_{10}(\eta )-( \gamma +m)f_{11}(\eta )] \Vert I_{i}(t)-I_{j}(t) \Vert , \\ \|T(A_{i}(t)-T(A_{j}(t))\| \leq [1+\delta \omega ^{\prime }_{p}f_{12}(\eta )-( \gamma ^{\prime }+m)f_{13}(\eta )] \Vert A_{i}(t)-A_{j}(t) \Vert , \\ \|T(R_{i}(t)-T(R_{j}(t))\| \leq [1+\gamma f_{14}(\eta )+\gamma ^{\prime } f_{15}( \eta )-mf_{16}(\eta )] \Vert R_{i}(t)-R_{j}(t) \Vert , \\ \|T(W_{i}(t)-T(W_{j}(t))\| \leq [1+\mu f_{17}(\eta )+\mu ^{\prime } f_{18}( \eta )-\varepsilon f_{19}(\eta )] \Vert W_{i}(t)-W_{j}(t) \Vert , \end{cases} $$

where

$$ \textstyle\begin{cases} (1-(m+\beta _{p}M_{3}+\beta _{p}M_{4}+\beta _{w}M_{6})f_{1}(\eta )- \beta _{p}M_{1}f_{2}(\eta )-\beta _{p}\kappa M_{1}f_{4}(\eta )-\beta _{w}M_{1}f_{4}( \eta ))< 1, \\ (1+\beta _{p}M_{1}f_{5}(\eta )+(\beta _{p}M_{3}+\beta _{p}\kappa M_{4}+ \beta _{w}M_{6})f_{6}(\eta )+\beta _{p}\kappa M_{1}f_{7}(\eta )+ \beta _{w}M_{1}f_{8}(\eta ) \\ \quad {}-((1-\delta )m+\delta \omega ^{\prime } +m)f_{9}(\eta ))< 1, \\ (1+(1-\delta )\omega f_{10}(\eta )-(\gamma +m)f_{11}(\eta ))< 1, \\ (1+\delta \omega ^{\prime }_{p}f_{12}(\eta )-(\gamma ^{\prime }+m)f_{13}(\eta ))< 1, \\ (1+\gamma f_{14}(\eta )+\gamma ^{\prime } f_{15}(\eta )-mf_{16}(\eta ))< 1, \\ (1+\mu f_{17}(\eta )+\mu ^{\prime } f_{18}(\eta )-\varepsilon f_{19}(\eta ))< 1. \end{cases} $$

Thus the *T*-self mapping has a fixed point. Also, we show that *T* satisfies the conditions in Theorem 4.1. Consider that (13), (14) hold, we assume

$$\begin{aligned}& B=(0,0,0,0,0,0), \\& b= \textstyle\begin{cases} (1-(m+\beta _{p}M_{3}+\beta _{p}M_{4}+\beta _{w}M_{6})f_{1}(\eta )- \beta _{p}M_{1}f_{2}(\eta )-\beta _{p}\kappa M_{1}f_{4}(\eta )-\beta _{w}M_{1}f_{4}( \eta )), \\ (1+\beta _{p}M_{1}f_{5}(\eta )+(\beta _{p}M_{3}+\beta _{p}\kappa M_{4}+ \beta _{w}M_{6})f_{6}(\eta )+\beta _{p}\kappa M_{1}f_{7}(\eta )+ \beta _{w}M_{1}f_{8}(\eta ) \\ \quad {}-((1-\delta )m+\delta \omega ^{\prime } +m)f_{9}(\eta )), \\ (1+(1-\delta )\omega f_{10}(\eta )-(\gamma +m)f_{11}(\eta )), \\ (1+\delta \omega ^{\prime }_{p}f_{12}(\eta )-(\gamma ^{\prime }+m)f_{13}(\eta )), \\ (1+\gamma f_{14}(\eta )+\gamma ^{\prime } f_{15}(\eta )-mf_{16}(\eta )), \\ (1+\mu f_{17}(\eta )+\mu ^{\prime } f_{18}(\eta )-\varepsilon f_{19}(\eta )). \end{cases}\displaystyle \end{aligned}$$

So, all the conditions of Theorem 4.1 are satisfied and the proof is complete. □

## Numerical method

In this section, we apply the homotopy analysis transform method (HATM) to implement the fractional model (1) appropriately. Notice that HATM is a well-developed mixture of the standard Laplace transform technique [45] and the homotopy analysis method (HAM) [46]. To solve model (1) by HATM, first we apply the Laplace transform in the following way:

$$ \textstyle\begin{cases} L[\frac{1}{\rho ^{1-\eta }} \,{}^{\mathrm{CF}}{D}^{\eta }_{t}S(t)](s)=L[\varLambda -mS(t)- \beta _{p}S(t)(I(t)+\kappa A(t))-\beta _{w}S(t)W(t)] , \\ L[\frac{1}{\rho ^{1-\eta }} \,{}^{\mathrm{CF}}{D}^{\eta }_{t}E(t)](s)=L[\beta _{p}S(t)(I(t)+ \kappa A(t))+\beta _{w}S(t)W(t) \\ \hphantom{L[\frac{1}{\rho ^{1-\eta }} \,{}^{\mathrm{CF}}{D}^{\eta }_{t}E(t)](s)={}}{}-(1-\delta )\omega E(t)-\delta \omega ^{\prime }E(t)-mE(t)] , \\ L[\frac{1}{\rho ^{1-\eta }} \,{}^{\mathrm{CF}}{D}^{\eta }_{t}I(t)](s)=L[(1-\delta ) \omega E(t)-(\gamma +m)I(t)] , \\ L[\frac{1}{\rho ^{1-\eta }} \,{}^{\mathrm{CF}}{D}^{\eta }_{t}A(t)](s)=L[\delta \omega ^{\prime }_{p}E(t)-(\gamma ^{\prime }+m)A(t)], \\ L[\frac{1}{\rho ^{1-\eta }} \,{}^{\mathrm{CF}}{D}^{\eta }_{t}R(t)](s)=L[\gamma I(t)+ \gamma ^{\prime }A(t)-mR(t)], \\ L[\frac{1}{\rho ^{1-\eta }} \,{}^{\mathrm{CF}}{D}^{\eta }_{t}W(t)](s)=L[\mu I(t)+ \mu ^{\prime }A(t)-\varepsilon W(t)], \end{cases} $$

which results in

$$ \textstyle\begin{cases} \frac{sL(S)-S(0)}{s+\eta (1-s)}=\rho ^{1-\eta } L[\varLambda -mS(t)- \beta _{p}S(t)(I(t)+\kappa A(t))-\beta _{w}S(t)W(t)] , \\ \frac{sL(E)-E(0)}{s+\eta (1-s)}=\rho ^{1-\eta } L[\beta _{p}S(t)(I(t)+ \kappa A(t))+\beta _{w}S(t)W(t) \\ \hphantom{\frac{sL(E)-E(0)}{s+\eta (1-s)}={}}{}-(1-\delta )\omega E(t)-\delta \omega ^{\prime }E(t)-mE(t)] , \\ \frac{sL(I)-I(0)}{s+\eta (1-s)}= \rho ^{1-\eta }L[(1-\delta )\omega E(t)-( \gamma +m)I(t)] , \\ \frac{sL(A)-A(0)}{s+\eta (1-s)}=\rho ^{1-\eta } L[\delta \omega ^{\prime }_{p}E(t)-( \gamma ^{\prime }+m)A(t)], \\ \frac{sL(R)-R(0)}{s+\eta (1-s)}=\rho ^{1-\eta } L[\gamma I(t)+\gamma ^{\prime }A(t)-mR(t)], \\ \frac{sL(W)-W(0)}{s+\eta (1-s)}=\rho ^{1-\eta } L[\mu I(t)+\mu ^{\prime }A(t)- \varepsilon W(t)]. \end{cases} $$

Then we get

15 $$ \textstyle\begin{cases} L(S)-\frac{S_{0}}{s}-\frac{s+\eta (1-s)}{s}\rho ^{1-\eta } L[\varLambda -mS(t)- \beta _{p}S(t)(I(t)+\kappa A(t))-\beta _{w}S(t)W(t)]=0 , \\ L(E)-\frac{E_{0}}{s}-\frac{s+\eta (1-s)}{s}\rho ^{1-\eta }L[\beta _{p}S(t)(I(t)+ \kappa A(t))+\beta _{w}S(t)W(t)-(1-\delta )\omega E(t) \\ \quad {}-\delta \omega ^{\prime }E(t)-mE(t)]=0 , \\ L(I)-\frac{I_{0}}{s}-\frac{s+\eta (1-s)}{s}\rho ^{1-\eta }L[(1- \delta )\omega E(t)-(\gamma +m)I(t)]=0 , \\ L(A)-\frac{A_{0}}{s}-\frac{s+\eta (1-s)}{s}\rho ^{1-\eta }L[\delta \omega ^{\prime }_{p}E(t)-(\gamma ^{\prime }+m)A(t)]=0, \\ L(R)-\frac{R_{0}}{s}-\frac{s+\eta (1-s)}{s}\rho ^{1-\eta }L[\gamma I(t)+ \gamma ^{\prime }A(t)-mR(t)]=0, \\ L(W)-\frac{W_{0}}{s}-\frac{s+\eta (1-s)}{s}\rho ^{1-\eta }L[\mu I(t)+ \mu ^{\prime }A(t)-\varepsilon W(t)]=0. \end{cases} $$

Using the homotopy method, we define

$$\begin{aligned}& N_{1}\bigl(\phi _{1}(t;q), \phi _{2}(t;q), \phi _{3}(t;q),\phi _{4}(t;q), \phi _{5}(t;q), \phi _{6}(t;q)\bigr) \\& \quad =L\bigl[\varLambda -m \phi _{1}(t;q)-\beta _{p}\phi _{1}(t;q)\phi _{3}(t;q)+ \kappa \phi _{4}(t;q))-\beta _{w}\phi _{1}(t;q)\phi _{6}(t;q)\bigr], \\& N_{2}\bigl(\phi _{1}(t;q), \phi _{2}(t;q), \phi _{3}(t;q),\phi _{4}(t;q), \phi _{5}(t;q), \phi _{6}(t;q)\bigr) \\& \quad =L\bigl[\beta _{p}\phi _{1}(t;q) \bigl(\phi _{3}(t;q)+\kappa \phi _{4}(t;q)\bigr)+ \beta _{w}\phi _{1}(t;q)\phi _{6}(t;q) \\& \qquad {}-(1-\delta )\omega \phi _{2}(t;q)- \delta \omega ^{\prime }\phi _{2}(t;q)-m\phi _{2}(t;q)\bigr], \\& N_{3}\bigl(\phi _{1}(t;q), \phi _{2}(t;q), \phi _{3}(t;q),\phi _{4}(t;q), \phi _{5}(t;q), \phi _{6}(t;q)\bigr) \\& \quad =L\bigl[(1-\delta )\omega \phi _{2}(t;q)-(\gamma +m)\phi _{3}(t;q)\bigr], \\& N_{4}\bigl(\phi _{1}(t;q), \phi _{2}(t;q), \phi _{3}(t;q),\phi _{4}(t;q), \phi _{5}(t;q), \phi _{6}(t;q)\bigr) \\& \quad =L\bigl[\delta \omega ^{\prime }_{p}\phi _{2}(t;q)- \bigl(\gamma ^{\prime }+m\bigr)\phi _{4}(t;q)\bigr], \\& N_{5}\bigl(\phi _{1}(t;q), \phi _{2}(t;q), \phi _{3}(t;q),\phi _{4}(t;q), \phi _{5}(t;q), \phi _{6}(t;q)\bigr) \\& \quad =L\bigl[\gamma \phi _{3}(t;q)+\gamma ^{\prime }\phi _{4}(t;q)-m\phi _{5}(t;q)\bigr], \\& N_{6}\bigl(\phi _{1}(t;q), \phi _{2}(t;q), \phi _{3}(t;q),\phi _{4}(t;q), \phi _{5}(t;q), \phi _{6}(t;q)\bigr) \\& \quad =L\bigl[\mu \phi _{3}(t;q)+\mu ^{\prime }\phi _{4}(t;q)-\varepsilon \phi _{6}(t;q)\bigr]. \end{aligned}$$

Then the deformation equations become

$$\begin{aligned}& (1-q)L\bigl[\phi _{1}(t;q)-S_{0}(t) \bigr] \\& \quad =qhH(t)N_{1}\bigl(\phi _{1}(t;q),\phi _{2}(t;q), \phi _{3}(t;q),\phi _{4}(t;q), \phi _{5}(t;q),\phi _{6}(t;q)\bigr), \\& (1-q)L\bigl[\phi _{2}(t;q)-E_{0}(t) \bigr] \\& \quad =qhH(t)N_{2}\bigl(\phi _{1}(t;q),\phi _{2}(t;q), \phi _{3}(t;q),\phi _{4}(t;q), \phi _{5}(t;q),\phi _{6}(t;q)\bigr), \\& (1-q)L\bigl[\phi _{3}(t;q)-I_{0}(t) \bigr] \\& \quad =qhH(t)N_{3}\bigl(\phi _{1}(t;q),\phi _{2}(t;q), \phi _{3}(t;q),\phi _{4}(t;q), \phi _{5}(t;q),\phi _{6}(t;q)\bigr), \\& (1-q)L\bigl[\phi _{4}(t;q)-A_{0}(t) \bigr] \\& \quad =qhH(t)N_{4}\bigl(\phi _{1}(t;q),\phi _{2}(t;q), \phi _{3}(t;q),\phi _{4}(t;q), \phi _{5}(t;q),\phi _{6}(t;q)\bigr), \\& (1-q)L\bigl[\phi _{5}(t;q)-R_{0}(t) \bigr] \\& \quad =qhH(t)N_{5}\bigl(\phi _{1}(t;q),\phi _{2}(t;q), \phi _{3}(t;q),\phi _{4}(t;q), \phi _{5}(t;q),\phi _{6}(t;q)\bigr), \\& (1-q)L\bigl[\phi _{6}(t;q)-W_{0}(t) \bigr] \\& \quad =qhH(t)N_{6}\bigl(\phi _{1}(t;q),\phi _{2}(t;q), \phi _{3}(t;q),\phi _{4}(t;q), \phi _{5}(t;q),\phi _{6}(t;q)\bigr), \end{aligned}$$

where $q\in [0,1]$ denotes an embedding parameter; $\phi _{i}(t; q)$, $i = 0, 1$, are unknown functions; $S_{0}$, $E_{0}$, $I_{0}$, $A_{0}$, $R_{0}$, $W_{0}$ are initial guesses; $L[\cdot]$ is the Laplace operator; $H(t)\neq 0$ is an auxiliary function, and $h \neq 0$ is a nonzero auxiliary parameter. Clearly, for $q= 0$ and $q = 1$, we have

$$ \textstyle\begin{cases} \phi _{1}(t;0)=S_{0}(t) ,\qquad \phi _{1}(t;1)=S(t), \\ \phi _{2}(t;0)=E_{0}(t) ,\qquad \phi _{2}(t;1)=E(t), \\ \phi _{3}(t;0)=I_{0}(t) ,\qquad \phi _{3}(t;1)=I(t), \\ \phi _{4}(t;0)=A_{0}(t) ,\qquad \phi _{4}(t;1)=A(t), \\ \phi _{5}(t;0)=R_{0}(t) ,\qquad \phi _{5}(t;1)=R(t), \\ \phi _{6}(t;0)=W_{0}(t) ,\qquad \phi _{6}(t;1)=W(t). \end{cases} $$

Thus, increasing *q* from zero to one varies the solution $(\phi _{1}(t;q),\phi _{2}(t;q),\phi _{3}(t;q), \phi _{4}(t;q), \phi _{5}(t;q), \phi _{6}(t;q))$ from $(S_{0}(t),E_{0}(t),I_{0}(t),A_{0}(t),R_{0}(t), W_{0}(t))$ to $(S(t),E(t),I(t),A(t),R(t), W(t))$. Now, we expand $\phi _{i}(t; q)$ ($i = 1, 2, 3, 4, 5, 6$) in the Taylor series with regard to *q*. This procedure yields

$$\begin{aligned}& \phi _{1}(t;q)=S_{0}+\sum_{n=1}^{\infty } S_{n}(t) q^{n},\qquad \phi _{2}(t;q)=E_{0}+ \sum_{n=1}^{\infty } E_{n}(t) q^{n}, \\& \phi _{3}(t;q)=I_{0}+\sum _{n=1}^{\infty } I_{n}(t) q^{n},\qquad \phi _{4}(t;q)=A_{0}+ \sum _{n=1}^{\infty } A_{n}(t) q^{n}, \\& \phi _{5}(t;q)=R_{0}+\sum _{n=1}^{\infty } R_{n}(t) q^{n},\qquad \phi _{6}(t;q)=W_{0}+ \sum _{n=1}^{\infty } W_{n}(t) q^{n}, \end{aligned}$$

where

16 $$ \begin{aligned} &S_{n}(t)= \frac{1}{n!} \frac{\partial ^{n}\phi _{1}(t;q)}{\partial q^{n}}\bigg| _{q=0},\qquad E_{n}(t)= \frac{1}{n!}\frac{\partial ^{n}\phi _{2}(t;q)}{\partial q^{n}}\bigg| _{q=0}, \\ &I_{n}(t)=\frac{1}{n!} \frac{\partial ^{n}\phi _{3}(t;q)}{\partial q^{n}}\bigg| _{q=0},\qquad A_{n}(t)= \frac{1}{n!} \frac{\partial ^{n}\phi _{4}(t;q)}{\partial q^{n}}\bigg| _{q=0}, \\ &R_{n}(t)=\frac{1}{n!} \frac{\partial ^{n}\phi _{5}(t;q)}{\partial q^{n}}\bigg| _{q=0},\qquad W_{n}(t)= \frac{1}{n!} \frac{\partial ^{n}\phi _{6}(t;q)}{\partial q^{n}}\bigg| _{q=0}. \end{aligned} $$

If the auxiliary function $H(t)$, the auxiliary parameter *h*, and the initial guesses are properly chosen, then series (16) converges at $q=1$, as proved by Liao [46]. Thus, we get

$$\begin{aligned}& S(t)=S_{0}+\sum_{n=1}^{\infty } S_{n}(t),\qquad E(t)=E_{0}+\sum_{n=1}^{ \infty } E_{n}(t), \\& I(t)=I_{0}+\sum_{n=1}^{\infty } I_{n}(t),\qquad A(t)=A_{0}+\sum_{n=1}^{ \infty } A_{n}(t), \\& R(t)=R_{0}+\sum_{n=1}^{\infty } R_{n}(t),\qquad W(t)=W_{0}+\sum_{n=1}^{ \infty } W_{n}(t). \end{aligned}$$

In addition, we can express the mth order deformation equation by

17 $$ \textstyle\begin{cases} L[S_{n}(t)-\chi _{n}S_{n-1}(t)]=hH T_{1,n}\vec{(S_{n-1})}, \\ L[E_{n}(t)-\chi _{n}E_{n-1}(t)]=hH T_{2,n}\vec{(E_{n-1})}, \\ L[I_{n}(t)-\chi _{n}I_{n-1}(t)]=hH T_{3,n}\vec{(I_{n-1})}, \\ L[A_{n}(t)-\chi _{n}A_{n-1}(t)]=hH T_{4,n}\vec{(A_{n-1})}, \\ L[R_{n}(t)-\chi _{n}R_{n-1}(t)]=hH T_{5,n}\vec{(R_{n-1})}, \\ L[W_{n}(t)-\chi _{n}W_{n-1}(t)]=hH T_{6,n}\vec{(W_{n-1})}, \end{cases} $$

where

18 $$ \textstyle\begin{cases} T_{1,n}(\vec{S_{n-1}}(t))=L[S_{n-1}(t)]-\frac{S_{0}}{s}(1-\chi _{n})- \frac{s+\alpha (1-s)}{s}\rho ^{1-\eta }L[\varLambda -mS_{n-1}(t) \\ \hphantom{T_{1,n}(\vec{S_{n-1}}(t)={}}{}-\beta _{p}S_{n-1}(t)(I_{n-1}(t)+\kappa A_{n-1}(t))-\beta _{w}S_{n-1}(t)W_{n-1}(t)], \\ T_{2,n}(\vec{E_{n-1}}(t))=L[E_{n-1}(t)]-\frac{E_{0}}{s}(1-\chi _{n}) \\ \hphantom{T_{2,n}(\vec{E_{n-1}}(t))={}}{}- \frac{s+\alpha (1-s)}{s}\rho ^{1-\eta }L[\beta _{p}S_{n-1}(t)(I_{n-1}(t)+ \kappa A_{n-1}(t)) \\ \hphantom{T_{2,n}(\vec{E_{n-1}}(t))={}}{}+\beta _{w}S_{n-1}(t)W_{n-1}(t)-(1-\delta )\omega E_{n-1}(t)-\delta \omega ^{\prime }E_{n-1}(t)-mE_{n-1}(t)], \\ T_{3,n}(\vec{I_{n-1}}(t))=L[I_{n-1}(t)] \\ \hphantom{T_{3,n}(\vec{I_{n-1}}(t))={}}{}-\frac{I_{0}}{s}(1-\chi _{n})- \frac{s+\alpha (1-s)}{s}\rho ^{1-\eta }L[(1-\delta )\omega E_{n-1}(t)-( \gamma +m)I_{n-1}(t)], \\ T_{4,n}(\vec{A_{n-1}}(t))=L[A_{n-1}(t)]-\frac{A_{0}}{s}(1-\chi _{n}) \\ \hphantom{T_{4,n}(\vec{A_{n-1}}(t))={}}{}- \frac{s+\alpha (1-s)}{s}\rho ^{1-\eta }L[\delta \omega ^{\prime }_{p}E_{n-1}(t)-( \gamma ^{\prime }+m)A_{n-1}(t)], \\ T_{5,n}(\vec{R_{n-1}}(t))=L[R_{n-1}(t)]-\frac{R_{0}}{s}(1-\chi _{n}) \\ \hphantom{T_{5,n}(\vec{R_{n-1}}(t))={}}{}- \frac{s+\alpha (1-s)}{s}\rho ^{1-\eta }L[\gamma I_{n-1}(t)+\gamma ^{\prime }A_{n-1}(t)-mR_{n-1}(t)], \\ T_{6,n}(\vec{W_{n-1}}(t))=L[W_{n-1}(t)]-\frac{W_{0}}{s}(1-\chi _{n}) \\ \hphantom{T_{6,n}(\vec{W_{n-1}}(t))={}}{}- \frac{s+\alpha (1-s)}{s}\rho ^{1-\eta }L[\mu I_{n-1}(t)+\mu ^{\prime }A_{n-1}(t)- \varepsilon W_{n-1}(t)], \end{cases} $$

and

$$ \chi _{n}= \textstyle\begin{cases} 0,& n\leq 1, \\ 1,& n>1. \end{cases} $$

Applying the inverse Laplace transform to equation (17), we obtain

$$\begin{aligned}& S_{n}(t)=\chi _{n}S_{n-1}(t)+hH L^{-1}\bigl[T_{1,n}(S_{n-1})\bigr],\qquad E_{n}(t)=\chi _{n}E_{n-1}(t)+hH L^{-1}\bigl[T_{2,n}(E_{n-1})\bigr], \\& I_{n}(t)=\chi _{n}I_{n-1}(t)+hH L^{-1}\bigl[T_{3,n}(I_{n-1})\bigr],\qquad A_{n}(t)=\chi _{n}A_{n-1}(t)+hH L^{-1}\bigl[T_{4,n}(A_{n-1})\bigr], \\& R_{n}(t)=\chi _{n}R_{n-1}(t)+hH L^{-1}\bigl[T_{5,n}(R_{n-1})\bigr],\qquad W_{n}(t)=\chi _{n}W_{n-1}(t)+hH L^{-1}\bigl[T_{6,n}(W_{n-1})\bigr]. \end{aligned}$$

Solving these equations for different values of $n = 1, 2, 3,\ldots$ , we derive

$$ \textstyle\begin{cases} S_{1}(t)=-hH \rho ^{1-\eta } (1+\alpha (t-1))(\varLambda -mS_{0}(t)- \beta _{p}S_{0}(t)(I_{0}(t)+\kappa A_{0}(t))-\beta _{w}S_{0}(t)W_{0}(t)) \\ \hphantom{S_{1}(t)}=-hHM_{1} \rho ^{1-\eta }(1+\alpha (t-1)), \\ E_{1}(t)=-hH \rho ^{1-\eta }(1+\alpha (t-1))(\beta _{p}S_{0}(t)(I_{0}(t)+ \kappa A_{0}(t))+\beta _{w}S_{0}(t)W_{0}(t) \\ \hphantom{E_{1}(t)={}}{}-(1-\delta )\omega E_{0}(t)-\delta \omega ^{\prime }E_{0}(t)-mE_{0}(t))=-hHM_{2} \rho ^{1-\eta }(1+ \alpha (t-1)), \\ I_{1}(t)=-hH \rho ^{1-\eta }(1+\alpha (t-1))((1-\delta )\omega E_{0}(t)-( \gamma +m)I_{0}(t)) \\ \hphantom{I_{1}(t)}=-hHM_{3} \rho ^{1-\eta }(1+\alpha (t-1)), \\ A_{1}(t)=-hH \rho ^{1-\eta }(1+\alpha (t-1))(\delta \omega ^{\prime }_{p}E_{0}(t)-( \gamma ^{\prime }+m)A_{0}(t)) \\ \hphantom{A_{1}(t)}=-hHM_{4} \rho ^{1-\eta }(1+\alpha (t-1)), \\ R_{1}(t)=-hH \rho ^{1-\eta }(1+\alpha (t-1))(\gamma I_{0}(t)+\gamma ^{\prime }A_{0}(t)-mR_{0}(t)) \\ \hphantom{R_{1}(t)}=-hHM_{5} \rho ^{1-\eta }(1+\alpha (t-1)), \\ W_{1}(t)=-hH \rho ^{1-\eta }(1+\alpha (t-1))(\mu I_{0}(t)+\mu ^{\prime }A_{0}(t)- \varepsilon W_{0}(t)) \\ \hphantom{W_{1}(t)}=-hHM_{6} \rho ^{1-\eta }(1+\alpha (t-1)), \end{cases} $$

where

$$ \textstyle\begin{cases} M_{1}=\varLambda -mS_{0}(t)-\beta _{p}S_{0}(t)(I_{0}(t)+\kappa A_{0}(t))- \beta _{w}S_{0}(t)W_{0}(t), \\ M_{2}=\beta _{p}S_{0}(t)(I_{0}(t)+\kappa A_{0}(t))+\beta _{w}S_{0}(t)W_{0}(t)-(1- \delta )\omega E_{0}(t)-\delta \omega ^{\prime }E_{0}(t)-mE_{0}(t), \\ M_{3}=(1-\delta )\omega E_{0}(t)-(\gamma +m)I_{0}(t), \\ M_{4}=\delta \omega ^{\prime }_{p}E_{0}(t)-(\gamma ^{\prime }+m)A_{0}(t), \\ M_{5}=\gamma I_{0}(t)+\gamma ^{\prime }A_{0}(t)-mR_{0}(t), \\ M_{6}=\mu I_{0}(t)+\mu ^{\prime }A_{0}(t)-\varepsilon W_{0}(t). \end{cases} $$

Finally, the solutions of system (1) are obtained as follows:

$$\begin{aligned}& S(t)=S_{0}(t)+S_{1}(t)+S_{2}(t)+\cdots, \\& E(t)=E_{0}(t)+E_{1}(t)+E_{2}(t)+\cdots, \\& I(t)=I_{0}(t)+I_{1}(t)+I_{2}(t)+\cdots, \\& A(t)=A_{0}(t)+A_{1}(t)+A_{2}(t)+\cdots, \\& R(t)=R_{0}(t)+R_{1}(t)+R_{2}(t)+\cdots, \\& W(t)=W_{0}(t)+W_{1}(t)+W_{2}(t)+\cdots. \end{aligned}$$

### Convergency of HATM for FDEs

In the following, we discuss the convergence of HATM by presenting and proving the following theorem.

#### Theorem 5.1

*Let*$\sum_{n=0}^{\infty }S_{n}(t)$, $\sum_{n=0}^{\infty }E_{n}(t)$, $\sum_{n=0}^{ \infty }I_{n}(t)$, $\sum_{n=0}^{\infty }A_{n}(t)$, $\sum_{n=0}^{\infty }R_{n}(t)$, *and*$\sum_{n=0}^{\infty }W_{n}(t)$*be uniformly convergent to*$S(t)$, $E(t)$, $I(t)$, $A(t)$, $R(t)$, *and*$W(t)$, *respectively*, *where*$\{S_{n}(t), E_{n}(t), I_{n}(t), A_{n}(t), R_{n}(t), W_{n}(t)\} \in L(R^{+})$*are produced by the mth order deformation* (17). *Also*, *assume that*$\sum_{n=0}^{\infty } ({}^{\mathrm{CF}}D^{\alpha }_{t}S_{n}(t))$, $\sum_{n=0}^{\infty } ({}^{\mathrm{CF}}D^{\alpha }E_{n}(t))$, $\sum_{n=0}^{\infty } ({}^{\mathrm{CF}}D^{\alpha }I_{n}(t))$, $\sum_{n=0}^{\infty } ({}^{\mathrm{CF}}D^{\alpha }A_{n}(t))$, $\sum_{n=0}^{\infty } ({}^{\mathrm{CF}}D^{\alpha }R_{n}(t))$, $\sum_{n=0}^{\infty } ({}^{\mathrm{CF}}D^{\alpha }W_{n}(t))$*are convergent*. *Then*$S(t)$, $E(t)$, $I(t)$, $A(t)$, $R(t)$, $W(t)$*are the exact solutions of system* (15).

#### Proof

By assuming that $\sum_{n=0}^{\infty }S_{n}(t)$ is uniformly convergent to $S(t)$, we can clearly state

19 $$ \lim_{n\rightarrow \infty }S_{n}(t)=0 ,\quad \mbox{for all } t\in R^{+}. $$

Since Laplace is a linear operator, we have

20 $$\begin{aligned}& \sum_{n=1}^{k}L \bigl[S_{n}(t)- \chi _{n}S_{n-1}(t)\bigr] \\& \quad =\sum _{n=1}^{k}\bigl[LS_{n}(t) -\chi _{n} LS_{n-1}(t)\bigr] \\& \quad = LS_{1}(t)+\bigl(LS_{2}(t)-LS_{1}(t) \bigr)+\cdots+\bigl(LS_{k}(t)-LS_{k-1}(t) \bigr)=LS_{k}(t). \end{aligned}$$

Thus, from (19) and (20) we derive

$$ \sum_{n=1}^{\infty }L \bigl[S_{n}(t)- \chi _{n}S_{n-1}(t)\bigr]=\lim_{k \rightarrow \infty }LS_{k}(t)=L \Bigl(\lim_{k\rightarrow \infty }S_{k}(t)\Bigr)=0. $$

Hence,

$$ hH\sum_{n=1}^{\infty } T_{1,n}\bigl( \vec{S}_{n-1}(t)\bigr)=\sum_{n=1}^{\infty }L \bigl[S_{n}(t)-\chi _{n}S_{n-1}(t)\bigr]=0. $$

Since $h\neq 0$, $H\neq 0$, this yields $\sum_{n=1}^{\infty } T_{1,n}(\vec{S}_{n-1}(t))=0$. Similarly, we can prove

$$\begin{aligned}& \sum_{n=1}^{\infty } T_{2,n}\bigl( \vec{E}_{n-1}(t)\bigr)=0,\qquad \sum_{n=1}^{\infty } T_{3,n}\bigl(\vec{I}_{n-1}(t)\bigr)=0, \\& \sum_{n=1}^{\infty } T_{4,n}\bigl( \vec{R}_{n-1}(t)\bigr)=0,\qquad \sum_{n=1}^{\infty } T_{5,n}\bigl(\vec{V}_{n-1}(t)\bigr)=0,\qquad \sum _{n=1}^{\infty } T_{6,n}\bigl( \vec{V}_{n-1}(t)\bigr)=0. \end{aligned}$$

Now, from (18) we get

$$\begin{aligned} 0 =&\sum_{n=1}^{\infty } \biggl\{ L \bigl[S_{n-1}(t)\bigr]-\frac{S_{0}}{s}(1-\chi _{n})- \frac{s+\alpha (1-s)}{s}\rho ^{1-\eta }L\bigl[\varLambda -nS_{n-1}(t) \\ &{}- \beta _{p}S_{n-1}(t) \bigl(I_{n-1}(t)+ \kappa A_{n-1}(t)\bigr)-\beta _{w}S_{n-1}(t)W_{n-1}(t)\bigr]\biggr\} \\ =&L\Biggl[\sum_{n=1}^{\infty }S_{n-1}(t) \Biggr]- \frac{S_{0}}{s}\sum_{n=1}^{\infty }(1- \chi _{n})- \frac{s+\alpha (1-s)}{s} \rho ^{1-\eta } L\Biggl[\sum _{n=1}^{\infty }\bigl( \varLambda -n S_{n-1}(t) \\ &{}-\beta _{p}S_{n-1}(t) \bigl(I_{n-1}(t)+\kappa A_{n-1}(t)\bigr)-\beta _{w}S_{n-1}(t)W_{n-1}(t) \bigr)\Biggr] \\ =& L\bigl[S(t)\bigr]-\frac{S_{0}}{s}-\frac{s+\alpha (1-s)}{s}\rho ^{1-\eta }L\bigl[ \varLambda -nS(t)-\beta _{p}S(t) \bigl(I(t)+ \kappa A(t)\bigr)-\beta _{w}S(t)W(t)\bigr]. \end{aligned}$$

Therefore $S(t)$ is the exact solution of system (15). Similarly, we can prove that $E(t)$, $I(t)$, $A(t)$, $R(t)$, and $W(t)$ are the exact solutions of system (15), and the proof is complete. □

## Numerical results

In this section, we present a numerical simulation for the transmission model of COVID-19 (1) by using the homotopy analysis transform method (HATM). To this end, we assume that the total population is $N=100$, and since the birth rate for China in 2020 is about 11.46 births per 1000 people, then $\varLambda =n\times N=1.146$. According to the news released by the World Health Organization, the death rate is 3.4 percent and the incubation period of COVID-19 is 14 days. Of course, the new Chinese study, which has yet to be peer-reviewed, suggests that the incubation period for the virus could be as long as 24 days.

Because the information is changing and due to the lack of complete information on many parameters related to the transmission of this virus, we had to consider some of the coefficients hypothetically. In this simulation, according to the news, we have chosen the parameters as $\beta _{p}=0.0025$, $\beta _{w}=0.001$, $\kappa =0.05$, $\delta =0.25$, $\omega =0.071$, $\omega ^{\prime }=0.1$, $\gamma =0.047$, $\gamma ^{\prime }=0.1$, $\mu =0.003$, $\mu ^{\prime }=0.001$, $\varepsilon =0.033$, and the initial values are $S_{0}=35$, $I_{0}=25$, $R_{0}=0$, $E_{0}=25$, $A_{0}=10$, $W_{0}=5$.

**Figure 1:**
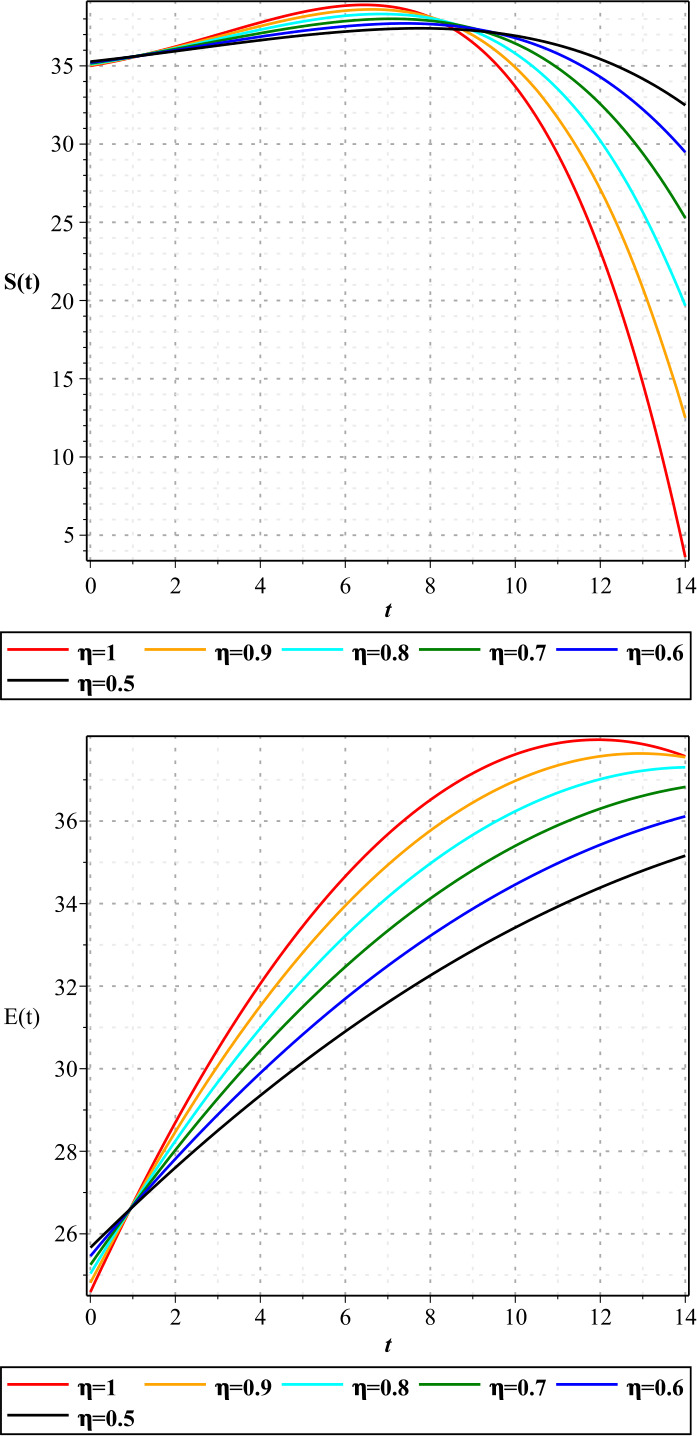
Plots of approximate solutions of susceptible parameter S and exposed parameter E for different values of $\eta =1,0.9,0.8,0.7,0.6,0.5$

**Figure 2:**
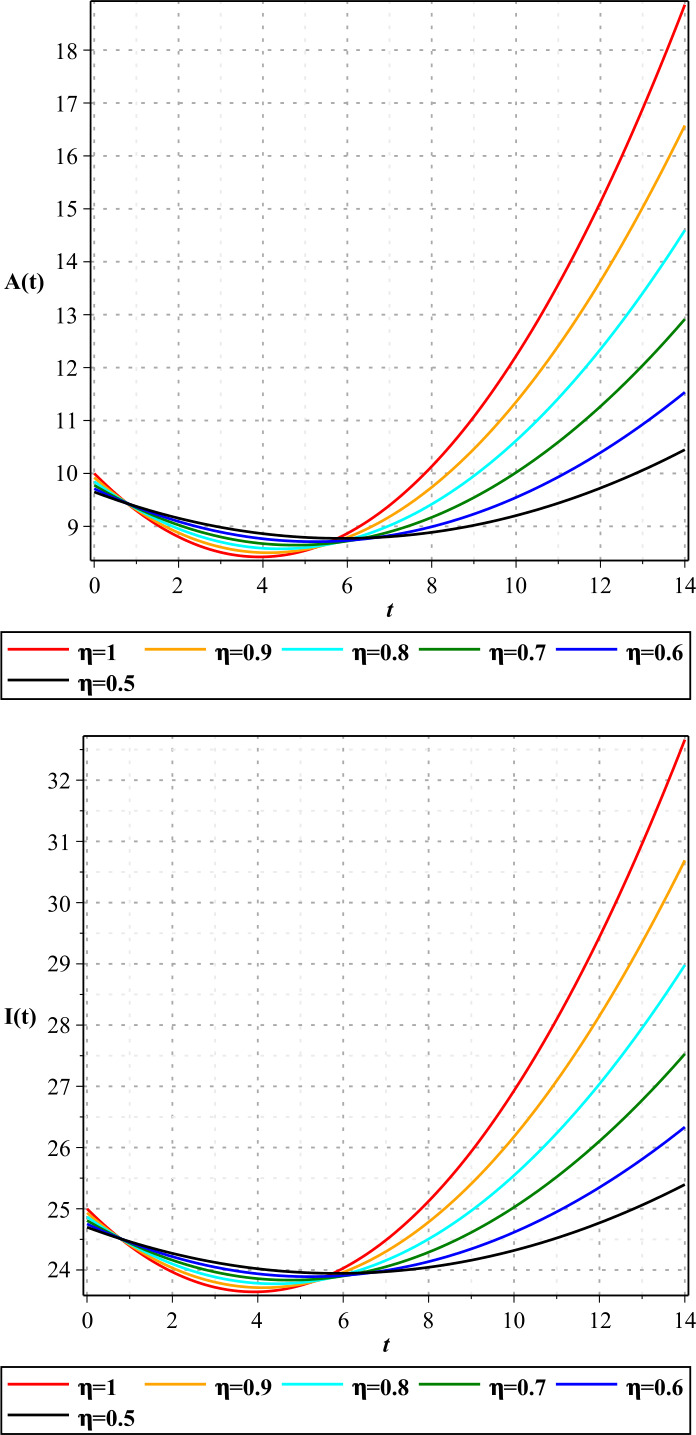
Plots of approximate solutions of asymptomatic infected parameter A and symptomatic infected parameter I for different values of $\eta =1,0.9,0.8,0.7,0.6,0.5$

**Figure 3:**
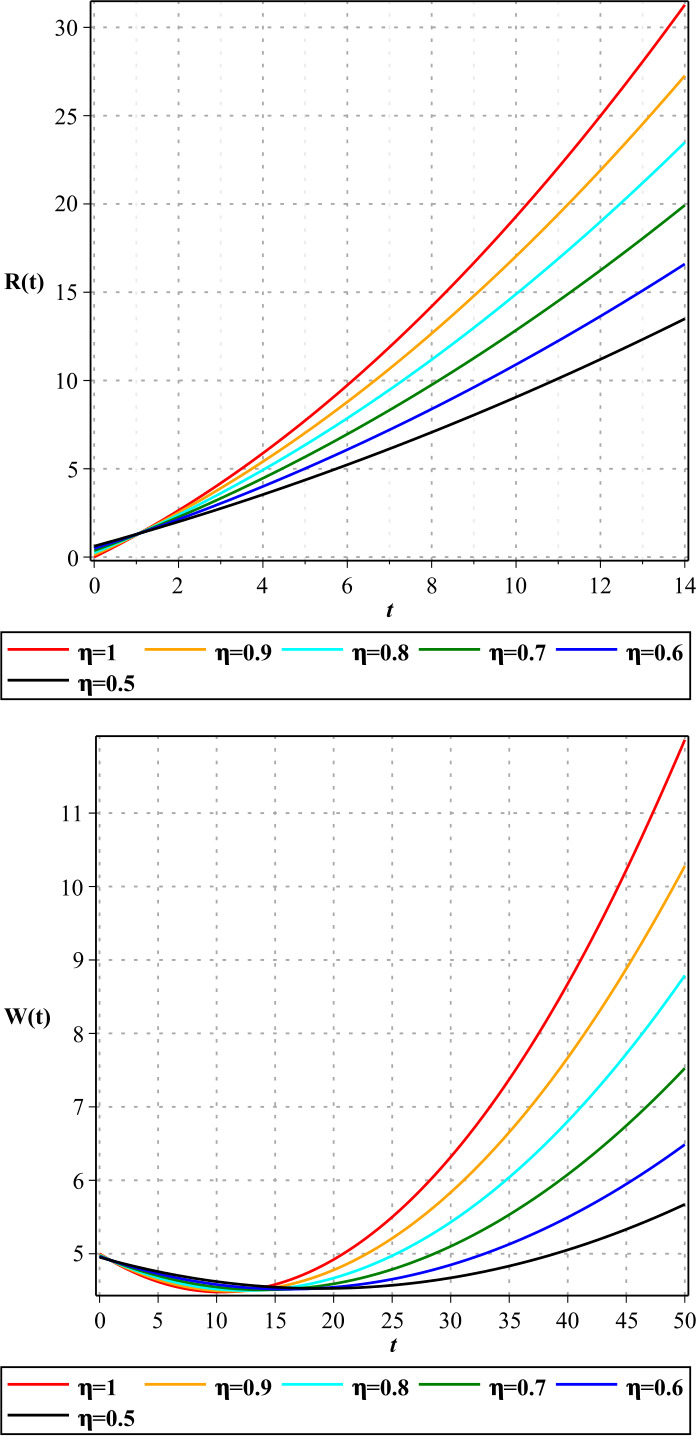
Plots of approximate solutions of removed parameter R and COVID-19 reservoir parameter W for different values of $\eta =1,0.9,0.8,0.7,0.6,0.5$

**Figure 4:**
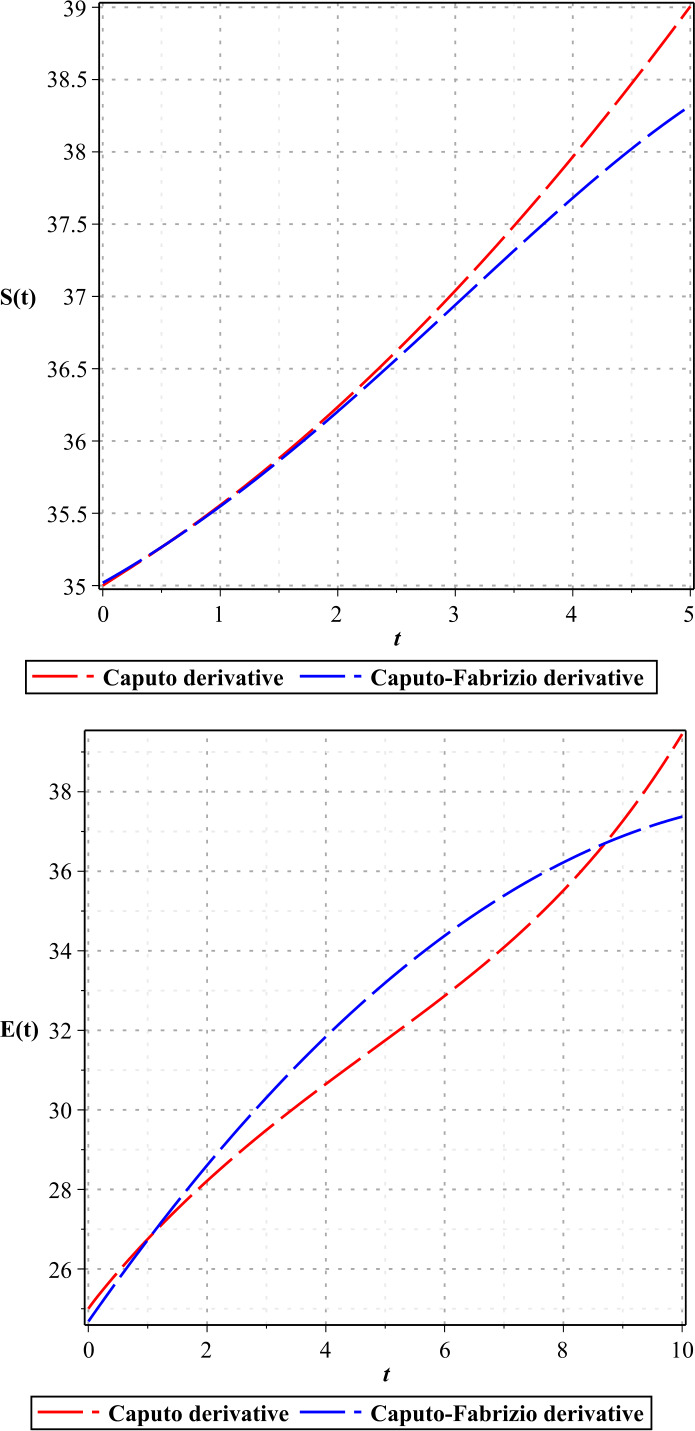
Plots of the results of Caputo derivative and Caputo–Fabrizio derivative for S, E with $\eta =0.96$

**Figure 5:**
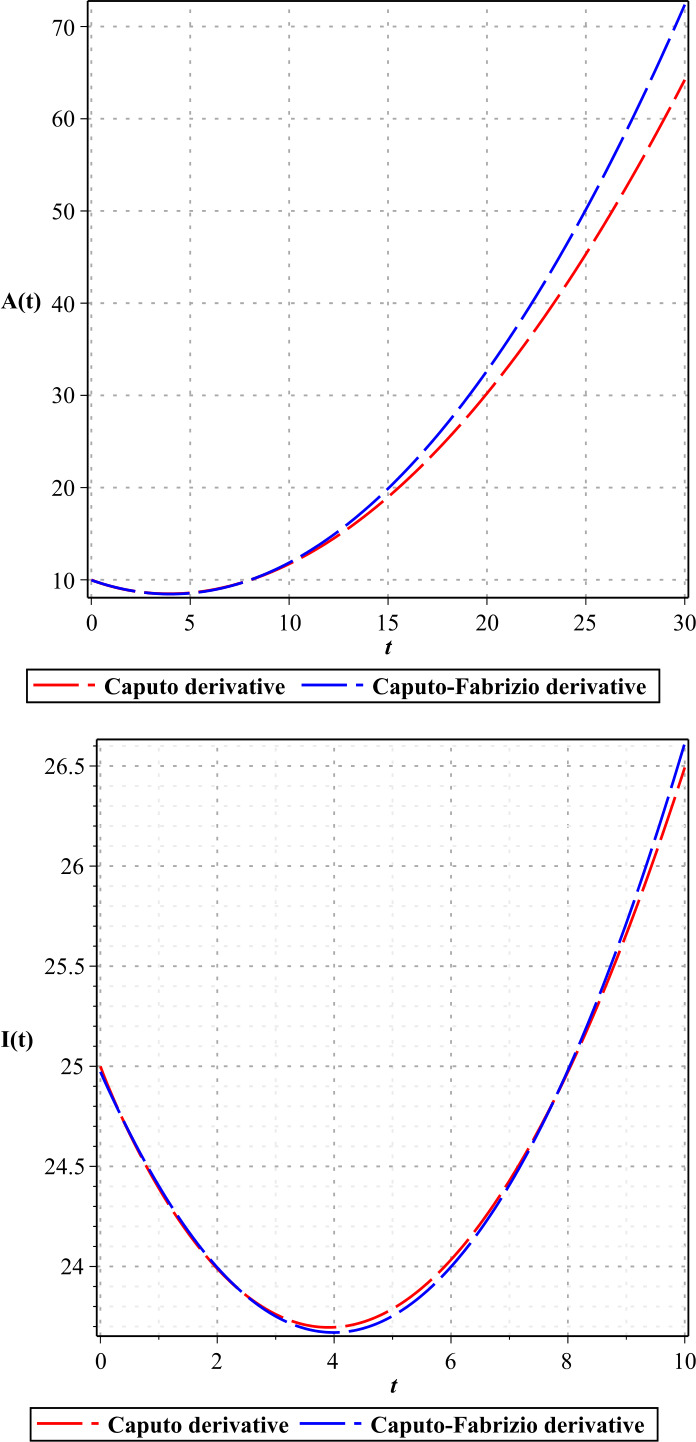
Plots of the results of Caputo derivative and Caputo–Fabrizio derivative for A, I with $\eta =0.96$

**Figure 6:**
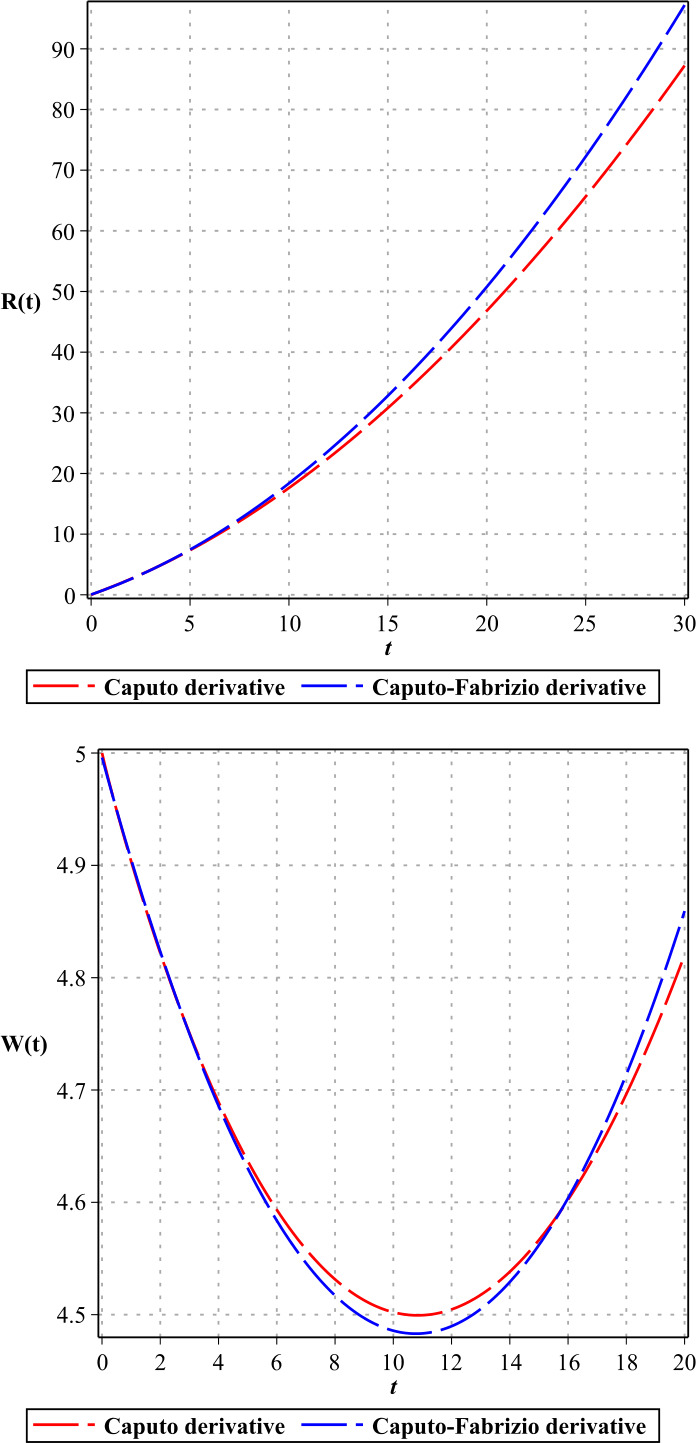
Plots of the results of Caputo derivative and Caputo–Fabrizio derivative for R, W with $\eta =0.96$

In Figures 1–3, we show the three-term solution of homotopy analysis transform method (HATM) with the auxiliary parameter $h = -1$ and the auxiliary function $H=1$ corresponding to proposed model (1) for different values of *η* and modification parameter $\rho =0.99$. Figures 1 and 2 show that the number of susceptible and exposed people increases first with a birth rate of 1.146. And then, with COVID-19 infection, the population of these two groups declines, and the population of the symptomatic and asymptomatic infected people increases. Figure 3 shows that the population of the out-group, i.e., the recovered and the dead, also increases with time. The amount of virus in the reservoir also decreases first and then increases as people enter the reservoir from the two infected groups. We put the Caputo fractional derivative in model (1) instead of the Caputo–Fabrizio fractional derivative and solved the new model similarly and obtained the results of the two derivatives for $\eta =0.96$. Then, in Figs. 4–6, we compared these results for system (1). We observe that the difference between the results of these two derivatives increases with time.

## Conclusion

In this paper, we investigate a model of the COVID-19 transmission in different groups of people using the Caputo–Fabrizio fractional derivative. Using the fixed point theorem, we prove a unique solution for the system. The resulting differential system is solved using the homotopy analysis transform method (HATM), and we obtain approximate solutions in convergent series. With the numerical results, we present a simulation for COVID-19, which shows the rapid transmission of the virus to different groups of people. We compared the results of the Caputo–Fabrizio fractional derivative with those of the Caputo derivative.
